# Inhibiting the miR-511-3p/PTCH1/Hedgehog Axis Alleviates Sepsis-Induced Systemic Inflammation and Acute Liver Injury

**DOI:** 10.3390/cells15212009

**Published:** 2026-11-04

**Authors:** Xi Deng, Haruka Kato, Eri Matsuo, Eiji Kawamoto, Motomu Shimaoka, Eun Jeong Park

**Affiliations:** Department of Molecular Pathobiology and Cell Adhesion Biology, Graduate School of Medicine, Mie University, Tsu 514-8507, Mie, Japan

**Keywords:** miR-511-3p, patched 1, hedgehog, sepsis, inflammation, intestinal epithelial cell, macrophage, liver

## Abstract

Sepsis is a life-threatening syndrome characterized by dysregulated systemic inflammation and multiple organ dysfunction, yet the molecular mechanisms underlying its progression remain incompletely understood. Our previous studies demonstrated that microRNA-511-3p (miR-511-3p) is upregulated in both an experimental sepsis model and patients with sepsis, suggesting its potential role in the pathogenesis of sepsis-associated inflammation. Because patched 1 (PTCH1), a validated target of miR-511-3p, serves as a key negative regulator of hedgehog (HH) signaling, we hypothesized that miR-511-3p promotes inflammatory responses during sepsis by suppressing PTCH1 expression and consequently activating HH signaling. To investigate this mechanism, we examined multiple immune cell populations, including small intestinal epithelial cells (S-IECs), small intestinal lamina propria macrophages (SI-LPMs), and liver macrophages (Liv-Ms), in a lipopolysaccharide (LPS)-induced murine sepsis model, as these cell populations may contribute to both local intestinal and systemic inflammatory responses during sepsis. The systemic administration of a miR-511-3p inhibitor (511-Inh) significantly reduced the expression of the proinflammatory cytokines interleukin 6 (IL-6) and tumor necrosis factor (TNF), as well as Hedgehog-interacting protein (HHIP), a canonical HH target gene. The inhibition of miR-511-3p restored PTCH1 expression in the intestines, liver, and lung and increased its mRNA levels in S-IECs, SI-LPMs, and Liv-Ms. These changes were accompanied by a reduced expression of HH pathway-related genes. Furthermore, miR-511-3p inhibition attenuated sepsis-induced liver injury, as evidenced by improved histopathological findings and decreased plasma levels of aspartate aminotransferase (AST) and alanine aminotransferase (ALT). To further determine the contribution of HH signaling to sepsis-associated inflammation, septic mice were treated with vismodegib, a selective inhibitor of smoothened (SMO). The pharmacological inhibition of SMO considerably reduced the expression of *Smo*, *Gli*, *Hhip*, *Il6*, and *Tnf* in the small intestine, large intestine, liver, and lung, confirming that aberrant HH pathway activation amplifies inflammatory responses during sepsis. Collectively, these findings suggest that miR-511-3p drives sepsis-associated inflammation, at least in part, by suppressing PTCH1 expression and thereby promoting HH signaling. Conversely, inhibiting miR-511-3p restores PTCH1 expression, dampens HH pathway activity, and mitigates systemic inflammation and sepsis-induced organ injury. Thus, the miR-511-3p/PTCH1/HH signaling axis may represent a promising therapeutic target for sepsis.

## 1. Introduction

Sepsis is a life-threatening syndrome caused by a dysregulated host response to infection and remains a leading cause of morbidity and mortality [1]. Each year, sepsis affects 49 million people and results in 11 million deaths, reaching a mortality rate of roughly 22% worldwide [2]. Despite substantial advances in intensive care unit (ICU) management, effective therapies that specifically target the molecular mechanisms driving sepsis are still lacking. Sepsis progresses through a massive release of inflammatory mediators, damage to body tissue, and dysfunction of multiple organs [3,4,5]. Although considerable efforts have been made to identify the signaling pathways responsible for uncontrolled inflammation, the regulatory mechanisms coordinating these responses remain incompletely understood.

The gastrointestinal tract is increasingly recognized as the motor of systemic inflammation during sepsis [6]. The intestinal epithelium forms the largest physical barrier and plays an essential role in preventing bacterial translocation and maintaining immune homeostasis [7]. During sepsis, intestinal barrier dysfunction allows microbial products, including LPS, to access the systemic circulation, thereby amplifying inflammatory responses in distant organs such as liver and lung [8,9,10]. In addition to intestinal epithelial cells (IECs), small intestinal lamina propria macrophages (SI-LPMs) are critical regulators of mucosal immunity [11,12]. While these macrophages contribute to intestinal homeostasis under physiological condition, they become activated and produce large amounts of inflammatory mediators during sepsis, thereby contributing to local and systemic inflammations [13]. However, the signaling pathways and their regulating factors activated in these cells during sepsis remain unknown.

MicroRNAs (miRNAs) are small non-coding RNAs that regulate gene expression in a post-transcriptional manner and have emerged as important modulators of innate and adaptive immune responses [14,15]. Several miRNAs have been proposed as biomarkers or therapeutic targets for sepsis [16,17,18,19,20]; however, the precise molecular mechanisms by which miRNAs regulate inflammatory signaling pathways remain to be fully elucidated. Patched 1 (PTCH1) is a validated target of miR-511-3p and serves as the inhibitory receptor of the hedgehog (HH) signaling pathway [21,22]. In the absence of HH ligands, PTCH1 suppresses the activity of smoothened (SMO), thereby preventing the activation of GLI transcription factors and HH target genes. Conversely, the loss or suppression of PTCH1 results in the constitutive activation of HH signaling. Although the HH pathway is well established in organ development and tissue regeneration [23], accumulating evidence indicates that aberrant HH signaling also regulates immune cell function, inflammatory cytokine production, and fibrotic responses [24,25,26,27,28]. Our previous findings showed that miR-511-3p is upregulated in both experimental sepsis models and patients with sepsis. Based on these observations, we hypothesized that increased miR-511-3p expression during sepsis suppresses PTCH1 expression, leading to the aberrant activation of HH signaling and upregulation of inflammatory responses.

In this study, we investigated the effects of systemic miR-511-3p inhibition in an LPS-induced murine model of sepsis. We focused particularly on intestinal epithelial cells (IECs), small intestinal lamina propria macrophages (SI-LPMs), and liver macrophages (Liv-Ms), which collectively propagate local and systemic inflammatory responses. Specifically, we examined whether miR-511-3p inhibition restores *Ptch1* expression, suppresses aberrant HH signaling activation, reduces proinflammatory cytokine production, and mitigates sepsis-induced organ injury. To further elucidate the role of the HH signaling pathway, we evaluated the selective SMO inhibitor vismodegib. Our findings identify the miR-511-3p/PTCH1/HH signaling axis as a previously unrecognized mechanism driving systemic inflammation during sepsis, suggesting that targeting this axis may offer a promising novel therapeutic strategy. Accordingly, this study aimed to validate systemic miR-511-3p inhibition as a therapeutic strategy in an acute model of sepsis by mitigating inflammatory responses and tissue injury. The primary outcomes were prespecified as the reduction in proinflammatory cytokine expression and the prevention of histopathological tissue injury after LPS administration.

## 2. Materials and Methods

### 2.1. Animal Models and Experimental Design

The animals used in this study were male and female C57BL/6J mice (Japan SLC, Shizuoka, Japan), aged 8–12 weeks at the start of the experiment, weighing 20 to 25 g. Mice were housed in the Mie University Experimental Animal Facilities under specific-pathogen-free conditions, with a 12 h light–dark cycle and ad libitum access to sterile food and water. To induce endotoxemia, mice received an intraperitoneal (i.p.) injection of lipopolysaccharide (LPS) (L3129, Sigma-Aldrich, St. Louis, MO, USA) at 5 mg/kg body weight in a volume of 0.2 mL of vehicle (phosphate-buffered saline; PBS). To inhibit miR-511-3p, mice were treated with 250 pmol of mmu-miR-511-3p synthetic tough decoy (S-TuD) dsRNA (GeneDesign, Osaka, Japan) via three i.p. injections at 2 h intervals, starting 2 h post-LPS administration. In some experiments, vismodegib (Selleck, Shizuoka, Japan) was administered i.p. using the same schedule (three injections of 125 nmol per injection). Three distinct experimental models were established: (1) LPS injection to healthy mice, (2) miR-511-3p inhibitor (511-Inh) dsRNA treatment in LPS-induced septic mice, and (3) vismodegib treatment in LPS-induced septic mice. Treatment groups were compared with the PBS vehicle, negative control (NC) dsRNA, and dimethyl sulfoxide (DMSO) vehicle-injected control group, respectively. Twenty-four hours after LPS injection, the mice were euthanized and their organs were harvested. This study used six groups of mice, with at least six mice per group for a total of more than forty mice. Animals were excluded from the experiment if they exhibited unexpected clinical sign within 24 h after treatment. All procedures were approved by the Ethics Review Committee for Animal Experimentation of Mie University (approval no. 2025-60 approved on 7 January 2022).

### 2.2. Clinical Samples

Serum was separated from the blood of healthy volunteers and patients via centrifugation at 3000 rpm for 15 min and stored in −80 °C freezer until use. The study protocols were approved by the Institutional Review Board of Mie University Hospital (approval nos. 3026; approved on 3 August 2016 and H2021-191 approved on 4 October 2021). Demographic and clinical data for the patients with sepsis are summarized in Table S1.

### 2.3. Isolation of Epithelial Cells (IECs) from Small Intestines

IECs were isolated from the small intestine (SI) and large intestine (LI) of C57BL/6J mice using a previously described method with minor modifications [29]. Briefly, intestinal tissues were flushed with RPMI-1640 medium (Nacalai Tesque, Kyoto, Japan) to remove luminal contents. Peyer’s patches were removed from the SI, and both SI and LI tissues were opened longitudinally and cut into approximately 1 cm segments. The tissue segments were shaken twice for 10 s in RPMI-1640 medium and subsequently incubated twice in RPMI-1640 with 2 mM EDTA and 5% fetal bovine serum (FBS) (Equitech-Bio, Kerrville, TX, USA) at 37 °C for 20 min with gentle stirring. The resulting single-cell suspensions were filtered through a 70 μm cell strainer (Corning, Glendale, AZ, USA) and centrifuged at 300× *g* for 5 min. The resulting cell pellets were used as the SI- and LI-derived cell fractions. For further enrichment of IECs, the remaining cell suspensions were layered onto a discontinuous 25%/40%/75% Percoll density gradient (GE Healthcare, Little Chalfont, UK) and centrifuged at 2000 rpm for 20 min in an AX-511 centrifuge (Tomy, Tokyo, Japan). The cell fraction at the 25%/40% Percoll interface was collected as the IEC-enriched fraction for subsequent analyses.

### 2.4. Isolation of Cells from Liver and Lung

Cells were isolated from liver, lung, and kidney tissues as previously described, with minor modifications [30]. Briefly, tissues were minced in RPMI-1640 supplemented with 5% FBS and passed through a 70 μm cell strainer (Corning) using the plunger of a syringe to obtain single-cell suspensions. The cell suspensions were centrifuged at 300× *g* for 5 min, and erythrocytes were subsequently lysed using ACK Lysing Buffer (Thermo Fisher Scientific). The remaining cells were collected by centrifugation at 300× *g* for 5 min, and the resulting pellets were stored at −80 °C until further analysis.

### 2.5. Isolation of CD11b^+^ Macrophages from Small-Intestinal and Liver Tissues

CD11b^+^ cells were isolated from small intestine (SI) and liver tissues using CD11b MicroBeads and magnetic-activated cell sorting (MACS) as previously described [31] with some modifications. Briefly, tissues were minced into small fragments using sterile scissors and digested in RPMI-1640 medium supplemented with 10% FBS and 2 mg/mL collagenase (FUJIFILM, Osaka, Japan). Enzymatic digestion was performed at 37 °C for three consecutive cycles of 10 min each under gentle agitation. The resulting cell suspensions were filtered through a 70 μm cell strainer to remove undigested tissue fragments and debris. CD11b^+^ cells were then enriched by MACS using CD11b MicroBeads (Miltenyi Biotec, Bergisch Gladbach, Germany) according to the manufacturer’s protocol.

### 2.6. Transient Transfection of a Human Epithelial Cell Line

A human epithelial cell line HCT116 was obtained from the American Type Culture Collection (ATCC, Manassas, VA, USA). Cells were cultured in McCoy’s 5A medium (Thermo Fisher Scientific, Waltham, MA, USA) supplemented with 10% FBS, 100 U/mL penicillin, and 100 μg/m streptomycin (Nacalai Tesque) and maintained in a humidified incubator at 37 °C under an atmosphere of 5% CO_2_. Cells were seeded onto 6-well plate (Corning, Glendale, AZ, USA) and grown to 70–80% confluency. To induce an inflammatory response, cells were stimulated with 1 μg/mL LPS (Sigma-Aldrich, St. Louis, MO, USA) for 6 h, after which the cells were washed and replenished with fresh medium. Either hsa-miR-511-3p antagomir (cat. no. 4464084, Thermo Fisher Scientific) or negative control (NC) RNA (Thermo Fisher Scientific) was transiently transfected into HCT116 cells using Lipofectamine RNAiMAX transfection reagent (Thermo Fisher Scientific) according to the manufacturer’s instructions. Transfections were carried out at a concentration of approximately 30 pmol of RNA antagomir per well in 6-well plates. Following transfection, the cells were cultured for 48 h before subsequent analyses.

### 2.7. Reverse Transcription Quantitative Polymerase Chain Reaction (RT-qPCR)

Total RNA was extracted from the cells or tissues using TRIzol reagent (Thermo Fisher Scientific) according to the manufacturer’s instructions. The reverse transcription of RNA was performed using the Mir-X miRNA First-Strand Synthesis Kit (Takara Bio, Shiga, Japan) for miRNA expression analysis or the PrimeScript RT Kit (Takara Bio) for mRNA expression analysis, following the manufacturers’ protocols. To evaluate relative gene expression, qPCR was conducted using the PowerUp SYBR Green Master Mix (Applied Biosystems, Foster City, CA, USA) on a QuantStudio 3 Real-Time PCR System (Thermo Fisher Scientific) according to the manufacturer’s instructions. For endogenous controls, the primers specific for *U6* small nuclear RNA and *β actin* were utilized to normalize the expression levels of miRNAs and mRNAs, respectively. For miRNA quantification, a universal primer (Thermo Fisher Scientific) was used for the validation runs. Relative gene expression levels were quantified using the comparative cycle threshold (Ct) method. For comparisons between experimental and control groups, relative expression was calculated using either 2^−ΔCT^ or 2^−ΔΔCT^ methods and expressed as fold change relative to the control group. In addition, expression levels normalized to the corresponding endogenous reference gene were calculated using the 2^−ΔCT^ method and presented as relative expression values. The primer sequences used in qPCR are listed in Table S2.

### 2.8. Western Blot Analysis

Intestinal, lung, and liver tissues harvested from LPS-induced septic mice treated with *511-Inh* or negative control (NC) were snap-frozen in liquid nitrogen and stored at −80 °C. Thawed tissues were homogenized using a pestle (Kenis, Osaka, Japan) and lysed in ice-cold Extraction Buffer (Abcam, Cambridge, UK) supplemented with protease inhibitor cocktail (Nacalai Tesque). Protein concentrations were determined using the Pierce Bicinchoninic Acid Protein Assay Kit (Thermo Fisher Scientific) on an iMark microplate reader (Bio-Rad, Hercules, CA, USA), according to the manufacturers’ instructions. Equal amounts of protein and a protein ladder (Nacalai Tesque) were loaded onto 7.5% precast gels (Bio-Rad) and separated by sodium dodecyl sulfate polyacrylamide gel electrophoresis (SDS-PAGE) using a Mini-PROTEAN Tetra Cell (Bio-Rad). The separated proteins were transferred onto polyvinylidene difluoride (PVDF) membrane (Cosmo Bio, Tokyo, Japan) using a Trans-Blot Transfer Cell (Bio-Rad). Membranes were blocked with 5% skim milk in Tris-buffered saline with Tween 20 (TBST) for 1 h at room temperature, followed by overnight incubation at 4 °C with primary antibodies against PTCH1 (A00441, Boster Biological Technology, Pleasanton, CA, USA), SMO (20787-1-AP, Proteintech, Rosemont, IL, USA), HHIP (29466-1-AP, Proteintech), or β actin (Sigma-Aldrich). After washing with TBST, the membranes were incubated with a horseradish peroxidase-conjugated secondary antibody (Abcam). Protein bands were visualized using an ECL Kit (Merck, Darmstadt, Germany) and captured using an ImageQuant LAS 4000mini imaging system (Cytiva, Tokyo, Japan).

### 2.9. Enzyme-Linked Immunosorbent Assay (ELISA)

Small-intestinal and liver tissue homogenates were prepared in ice-cold Cell Extraction Buffer (Abcam) according to the manufacturer’s instructions. Homogenates were incubated on ice and centrifuged to remove insoluble material, and the resulting supernatants were collected for analysis. Tissue concentrations of IL-6 and TNF were determined using mouse ELISA kits (ab222503 and ab208348, respectively, Abcam) according to the manufacturer’s protocols. Plasma levels of aspartate aminotransferase (AST) and alanine aminotransferase (ALT) were determined using mouse ELISA kits (ab263882 and ab282882, respectively, Abcam). Absorbance was measured at 450 nm using an iMark microplate reader (Bio-Rad), and analyte concentrations were calculated from the corresponding standard curves.

### 2.10. Histological Analysis

The liver, lung, kidney, and intestines were harvested from LPS-induced septic mice treated with either 511-Inh RNA or NC RNA. The collected tissue samples were fixed in 4% paraformaldehyde solution (Nacalai Tesque) overnight, washed with phosphate-buffered saline, and then delivered to the Genostaff (Tokyo, Japan) for histological analysis. Histological images were exported using NDP.view2 software (Hamamatsu Photonics, Shizuoka, Japan). Quantitative histological assessment was performed as previously described [32] with slight modifications. Sinusoidal congestion, vacuolization, necrosis, and inflammation were scored on 5-point scale (0, none; 1, minimal; 2, mild; 3, moderate; and 4, severe).

### 2.11. Statistical Analysis

Data were presented as mean and standard errors of the mean (SEM) and were analyzed using GraphPad Prism software (version 8.4.3, GraphPad, San Diego, CA, USA). Differences between the two groups were evaluated using an unpaired, two-tailed Student’s *t*-test. A *p* value less than 0.05 was considered statistically significant.

## 3. Results

### 3.1. Inflammatory Responses Are Associated with miR-511-3p Expression in IECs and Macrophages in Sepsis

**Figure 1:**
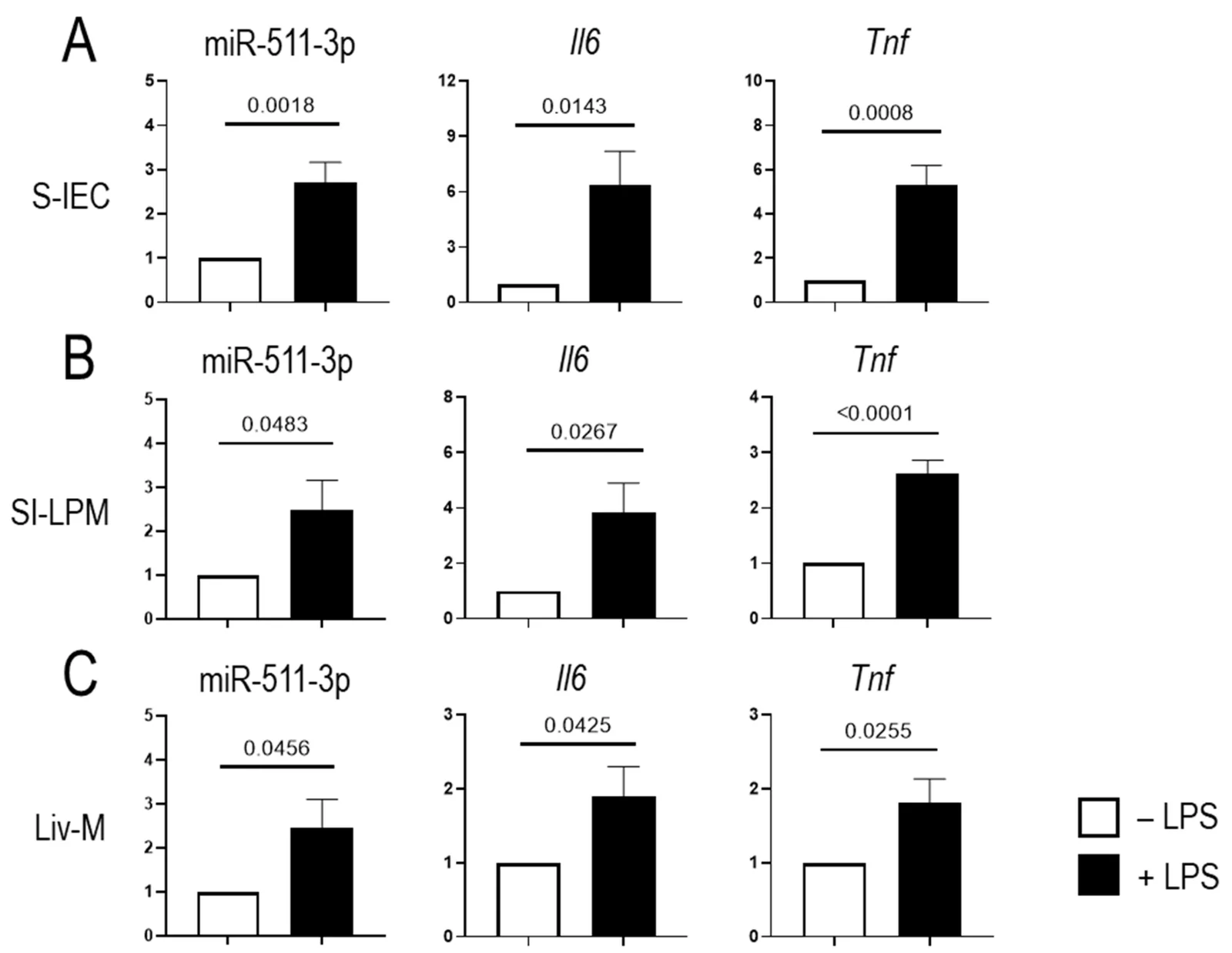
LPS treatment upregulates miR-511-3p, *Il6* and *Tnf* expressions across multiple cell populations. Relative gene expression levels in small intestinal epithelial cells (S-IECs) (**A**), small intestinal lamina propria macrophages (SI-LPMs) (**B**), and liver macrophages (Liv-Ms) (**C**) from control (- LPS) and LPS-treated (+ LPS) mice. Levels of miRNA and mRNA were quantified by RT-qPCR, normalized to *U6* and *Actb* (β-actin), respectively, and calculated using the 2^−ΔΔCT^ method relative to the control mean. Data represent mean ± SEM (*n* = 5–8 per group); *p*-values are displayed on all graphs, where *p* < 0.05 indicates a statistical significance. *Il6*, interleukin 6 gene; and *Tnf*, tumor necrosis factor gene.

In our previous study, we identified miR-511-3p as the only miRNA upregulated in both intestinal epithelial cells (IECs) and blood samples from a cecal slurry-induced chronic sepsis mouse model [33]. Consistently, serum miR-511-3p levels were also significantly elevated in patients with sepsis (*n* = 10) compared to healthy volunteers (*n* = 10) (Figure S1). Based on these observations, we hypothesized that miR-511-3p may contribute to the pathogenesis of sepsis during its acute phase. To test this possibility, we established an LPS-induced murine model of sepsis and evaluated miR-511-3p expression across major organs. LPS administration upregulated the expression of miR-511-3p (Figure S2A), as well as the proinflammatory cytokine genes *Il6* and *Tnf* (Figure S2B,C), in the small and large intestines, liver, and lung. Consistent with our previous findings in the chronic sepsis model [33], miR-511-3p expression was markedly elevated in small intestinal epithelial cells (S-IECs) from LPS-treated mice (Figure 1A). This increase was accompanied by enhanced expression of *Il6* and *Tnf* (Figure 1A), suggesting a potential association between miR-511-3p upregulation and inflammatory responses in IECs during sepsis.

Although miR-511-3p is upregulated in IECs during sepsis, this miRNA is encoded within intron 5 of the mannose receptor c-type 1 (Mrc1/CD206) gene and is co-expressed with CD206 [34]. Under basal conditions, miR-511-3p expression levels in CD11b^+^ macrophages isolated from the SI were 100-fold higher than those in IECs (Figure S3). Therefore, we investigated whether macrophages upregulate miR-511-3p in response to septic challenge. CD11b^+^ macrophages were isolated from the liver as well as the SI lamina propria of LPS-treated mice to evaluate miR-511-3p expression. Compared with mock-treated controls, small intestinal lamina propria macrophages (SI-LPMs) from septic mice exhibited considerably increased miR-511-3p expression, which were accompanied by the elevated expression of *Il6* and *Tnf* (Figure 1B). A similar positive correlation between miR-511-3p and proinflammatory cytokine expression was observed in liver macrophages (Liv-Ms) (Figure 1C). Both macrophages and IECs may contribute to the circulating pool of miR-511-3p, potentially enhancing the systemic inflammatory response during sepsis. Given that SI-LPMs exhibited higher miR-511-3p expression levels than IECs (Figure S3), they might represent a primary quantitative source of circulating miR-511-3p. Together, these findings indicate that miR-511-3p upregulation is closely associated with inflammatory activation across multiple organs affected by sepsis.

### 3.2. miR-511-3p Induction During Sepsis Is Associated with Activation of the Hedgehog Signaling Pathway

Given the robust induction of miR-511-3p in sepsis target organs and inflammatory cells, including IECs and macrophages, during sepsis, we next sought to identify downstream pathways regulated by this miRNA. Bioinformatic analyses using TargetScan and miRDB, together with previous experimental evidence [22,33], identified patched 1 (PTCH1) as a potential target of miR-511-3p. PTCH1 is a well-established negative regulator of the HH signaling pathway, which plays critical roles in tissue repair, immune regulation, and intestinal homeostasis [21,35,36]. Based on these notes, we hypothesized that the sepsis-induced upregulation of miR-511-3p may enhance HH signaling by repressing *Ptch1* expression. To test this, we examined the expression of *Ptch1* and downstream HH pathway component genes in septic tissues and cells.

**Figure 2:**
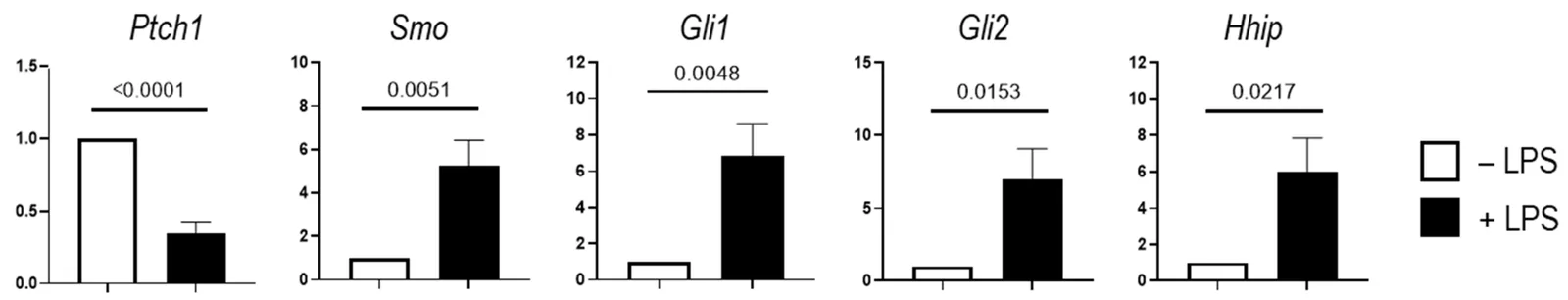
LPS treatment decreases *Ptch1* and increases *Smo*, *Gli1*, *Gli2*, and *Hhip* expressions in S-IECs. Relative gene expression levels in control (−LPS) and + LPS mice. Levels of mRNA were quantified by RT-qPCR, normalized to *Actb*, and calculated using the 2^−ΔΔCT^ method relative to the control mean. Data represent mean ± SEM (*n* = 5–9 per group). *Ptch1*, patched 1 gene; *Smo*, smoothened gene; *Gli*, glioma gene; *Hhip*, Hedgehog-interacting protein gene.

**Figure 3:**
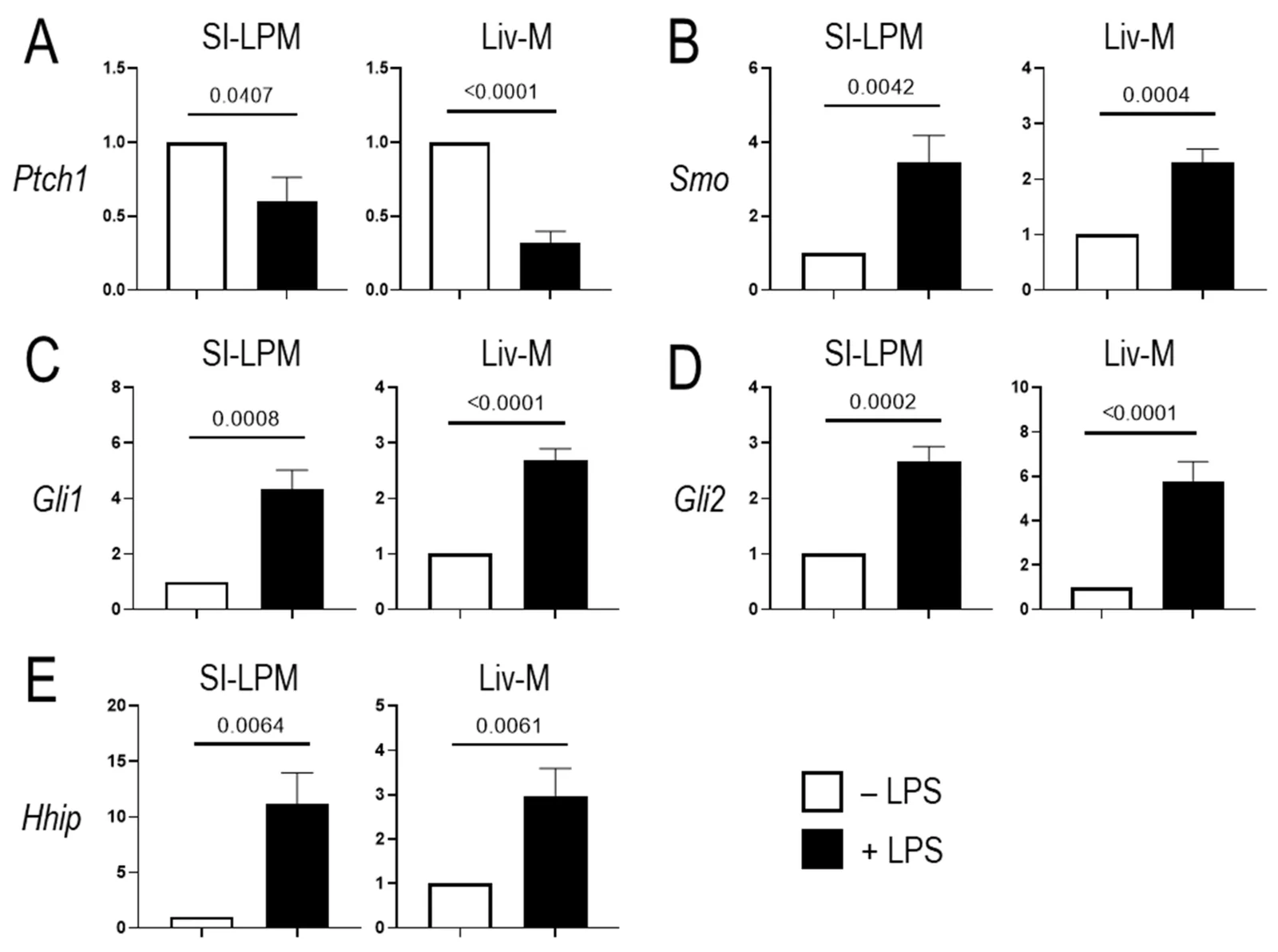
LPS treatment decreases expressions of *Ptch1* (**A**) and increased *Smo* (**B**), *Gli1* (**C**), *Gli2* (**D**), and *Hhip* (**E**) expressions. Relative gene expression levels in SI-LPMs and Liv-M from control (−LPS) and + LPS mice. Levels of mRNA were quantified by RT-qPCR, normalized to *Actb*, and calculated using the 2^−ΔΔCT^ method relative to the control mean. Data are represented as the mean ± SEM (*n* = 5–9 per group).

*Ptch1* expression was remarkably reduced in SI, LI, liver, and lung from LPS-treated mice, when compared with controls (Figure S4A). Consistent with the downregulation of *Ptch1*, LPS administration increased the expression of the HH signaling component genes, *Smo*, *Gli1*, *Gli2*, and *Hhip*, in SI, LI, liver, and lung (Figure S4B–E). Similar change in these HH genes was reproduced in S-IECs isolated from septic mice (Figure 2). Thus, an inverse correlation between miR-511-3p and *Ptch1* expression levels was detected, whereas miR-511-3p expression positively correlated with genes of HH components and proinflammatory cytokines. To determine whether these alterations also occurred in macrophages, we analyzed the SI-LPMs and Liv-Ms. Similar to our observations in S-IECs, septic macrophages from both organs displayed reduced *Ptch1* expression alongside enhanced expression of HH pathway-related genes (Figure 3A–E). These findings suggest that HH pathway activation is a common response in S-IECs and macrophages during sepsis and is closely linked to the induction of inflammation. Specifically, the sepsis-induced upregulation of miR-511-3p correlates with the downregulation of the HH pathway suppressor *Ptch1*, driving subsequent HH activation. These observations prompted us to investigate whether inhibiting miR-511-3p could ameliorate septic inflammation and tissue damage by restoring *Ptch1* expression and suppressing HH signaling.

### 3.3. Inhibition of miR-511-3p Attenuates Systemic Inflammation in LPS-Induced Sepsis

**Figure 4:**
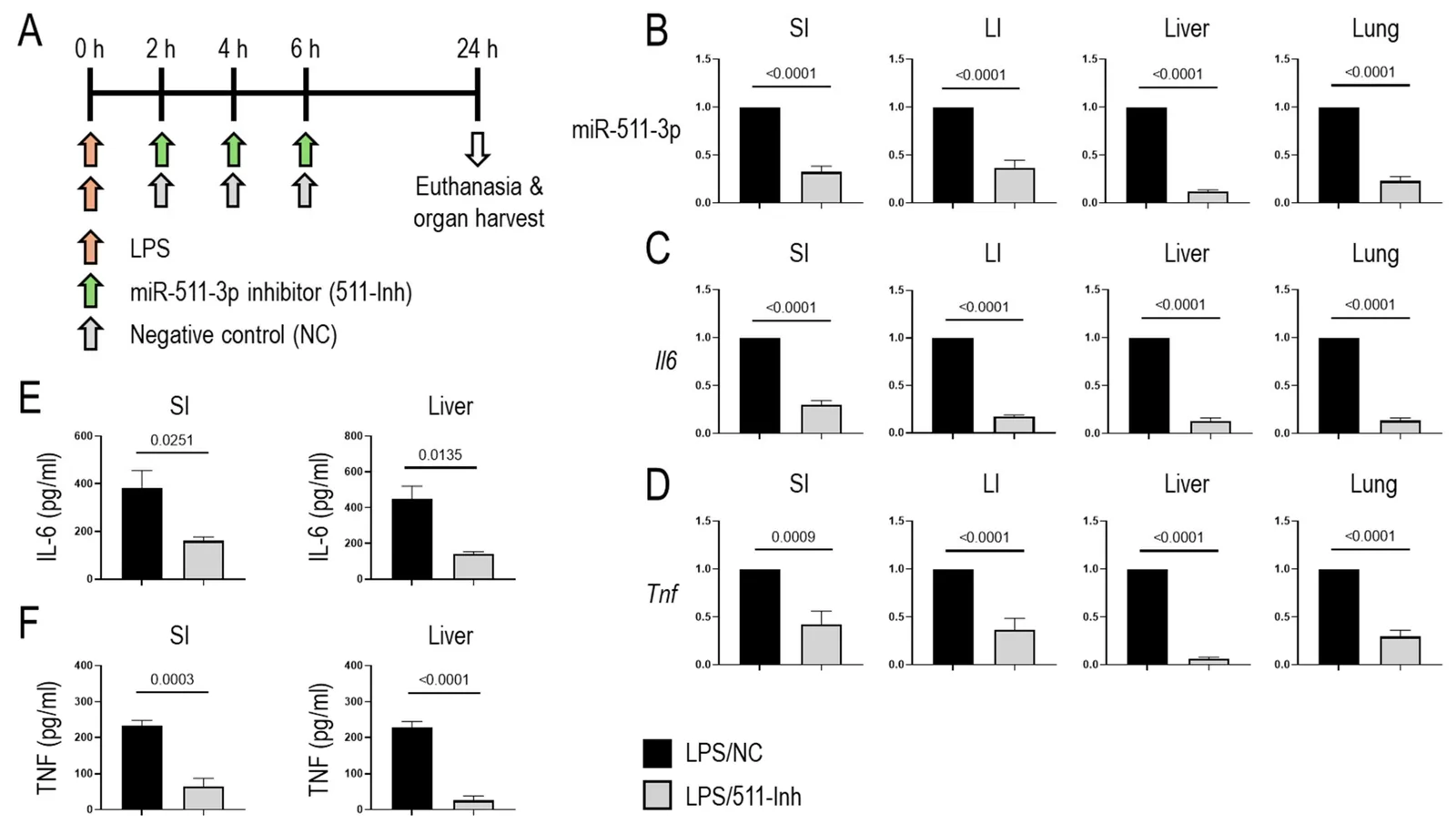
miR-511-3p inhibitor RNA (511-Inh) attenuates inflammatory responses in LPS-induced septic mice. (**A**) Experimental design timeline. (**B**–**D**) Relative gene expression levels in small intestine (SI), large intestine (LI), liver, and lung from LPS-induced sepsis mice treated with negative control RNA (LPS/NC) or 511-Inh (LPS/511-Inh). Levels of miRNA and mRNA were quantified by RT-qPCR, normalized to *U6* and *Actb* (β-actin), respectively, and calculated using the 2^−ΔΔCT^ method relative to the control mean. (**E**,**F**) Protein levels of IL-6 (**E**) and TNF (**F**) in SI and liver tissues were measured by ELISA. Data are presented as the mean ± SEM (*n* = 5–11 per group for gene expression and *n* = 4–5 per group for ELISA).

**Figure 5:**
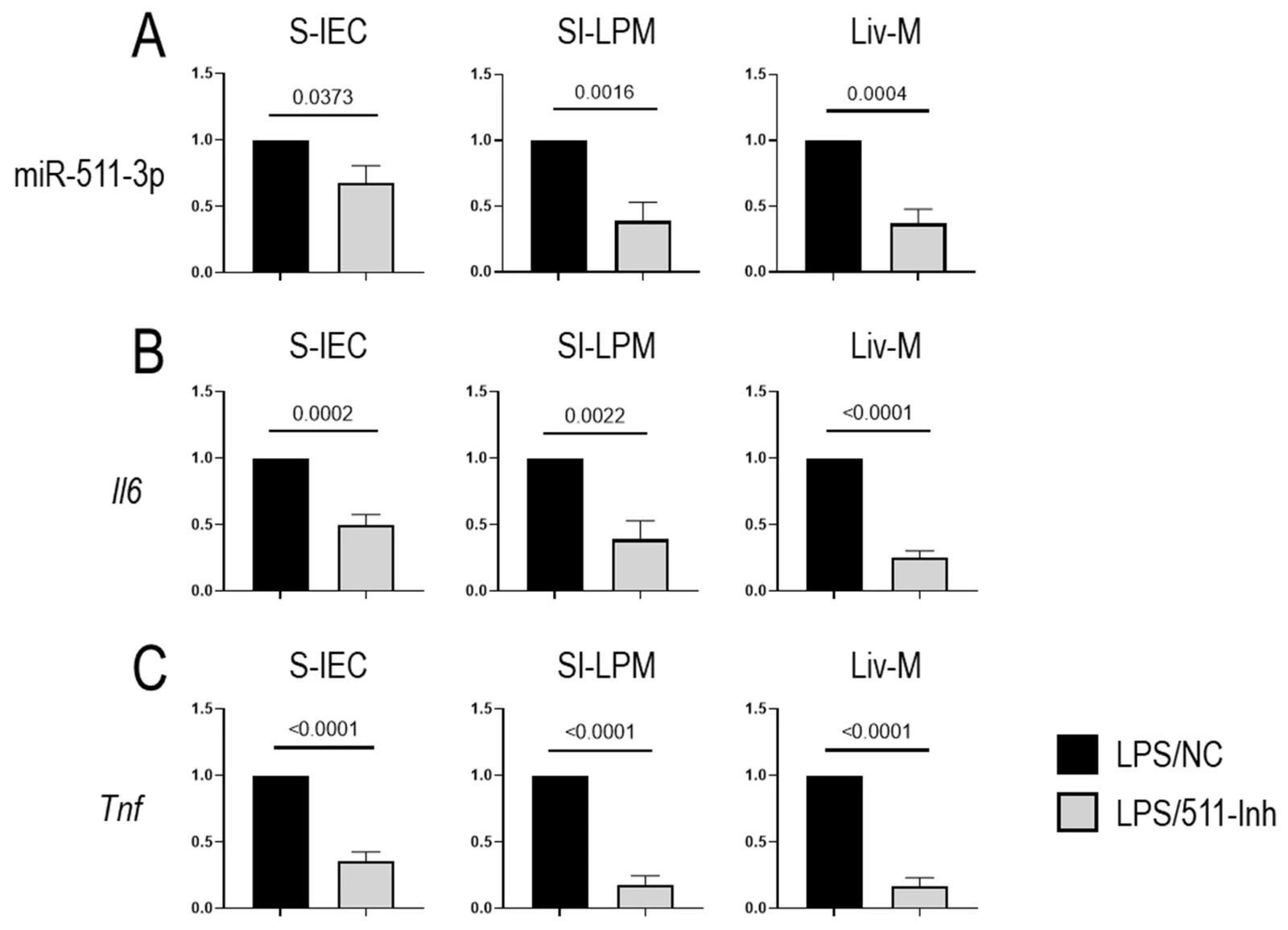
511-Inh reduces miR-511-3p, *Il6* and *Tnf* expressions in LPS-induced septic mice. Relative gene expression levels in S-IECs (**A**), SI-LPMs (**B**), and Liv-Ms (**C**) from LPS/NC or LPS/511-Inh mice. Levels of miRNA and mRNA were quantified by RT-qPCR, normalized to *U6* and *Actb* (β-actin), respectively, and calculated using the 2^−ΔΔCT^ method relative to the control mean. Data represent mean ± SEM (*n* = 5–6 per group).

To determine whether elevated miR-511-3p expression contributes to sepsis-associated inflammation, mice with LPS-induced sepsis were treated with a miR-511-3p inhibitor (511-Inh) consisting a specific synthetic tough decoy (S-TuD), dsRNA (Figure 4A). Compared with the NC group, three doses of 250 pmol 511-Inh suppressed miR-511-3p expression by 63% to 87% across all tested tissues (Figure 4B). This robust downregulation demonstrates that the administered dose is highly effective for the systemic in vivo knockdown of miR-511-3p. Concomitantly, the mRNA expression levels of *Il6* and *Tnf* were downregulated in SI, LI, liver, and lung of septic mice treated with 511-Inh compared with those treated with the negative control (NC) dsRNA (Figure 4C,D). Consistent with these findings, protein-level analyses confirmed significant reductions in IL-6 and TNF in SI and liver tissues of 511-Inh-treated septic mice (Figure 4E,F). At the cellular level, miR-511-3p inhibition was associated with reductions in *Il6* expression by 50–75% and *Tnf* expression by 64–83% across S-IECs, SI-LPMs and Liv-Ms (Figure 5A–C). Collectively, these findings indicate that miR-511-3p promotes inflammatory responses across multiple cell populations and target organs during sepsis.

**Figure 6:**
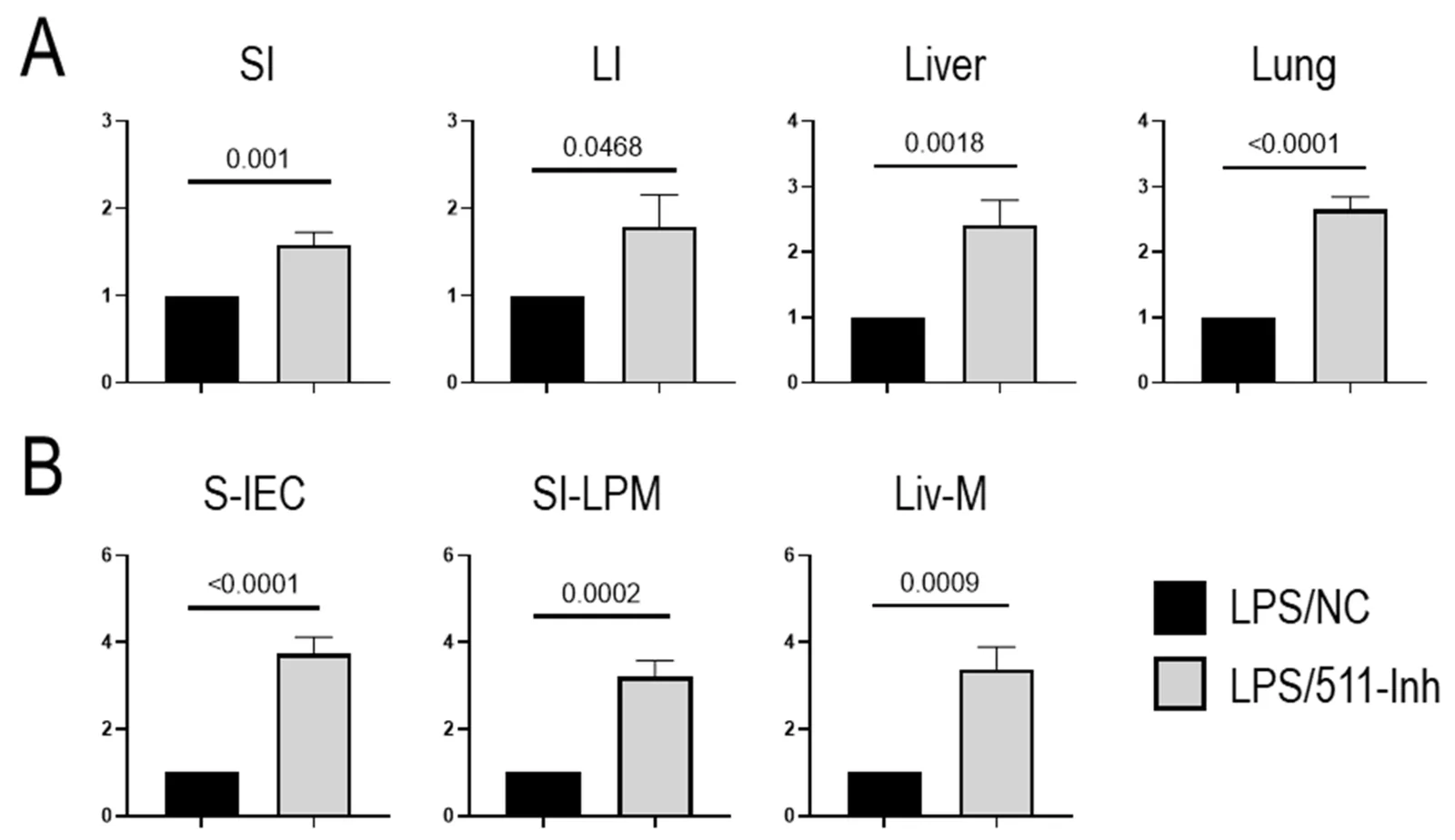
511-Inh increases *Ptch1* expression across multiple tissues (**A**) and three cell populations (**B**) in LPS-induced septic mice. Relative gene expression levels in SI, LI, liver, and lung (**A**) and S-IECs, SI-LPMs, and Liv-Ms (**B**) from LPS/NC or LPS/511-Inh mice. Levels of *Ptch1* mRNA were quantified by RT-qPCR, normalized to *Actb*, and calculated using the 2^−ΔΔCT^ method relative to the control mean. Data represent mean ± SEM (*n* = 5–11 per group).

**Figure 7:**
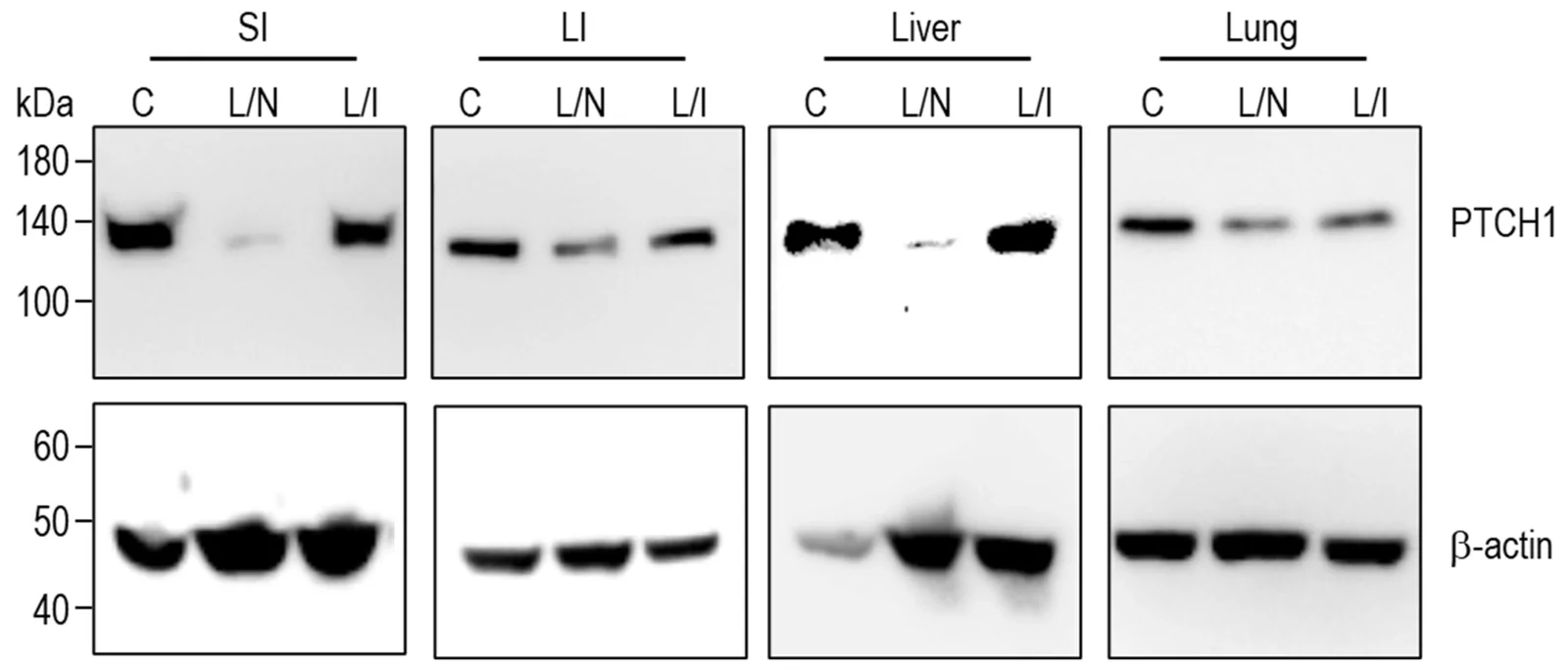
511-Inh treatment restores PTCH1 protein expression across multiple tissues, including SI, LI, liver, and lung. Representative Western blot images using anti-PTCH1 (**upper**) and anti-β-actin antibodies (**lower panel**) are shown. For each tissue, the three lanes represent lysates from each tissue of untreated wild-type control mice (C), LPS-induced septic mice treated with NC (L/N), and LPS-induced septic mice treated with 511-Inh (L/I). kDa, kilodalton.

**Figure 8:**
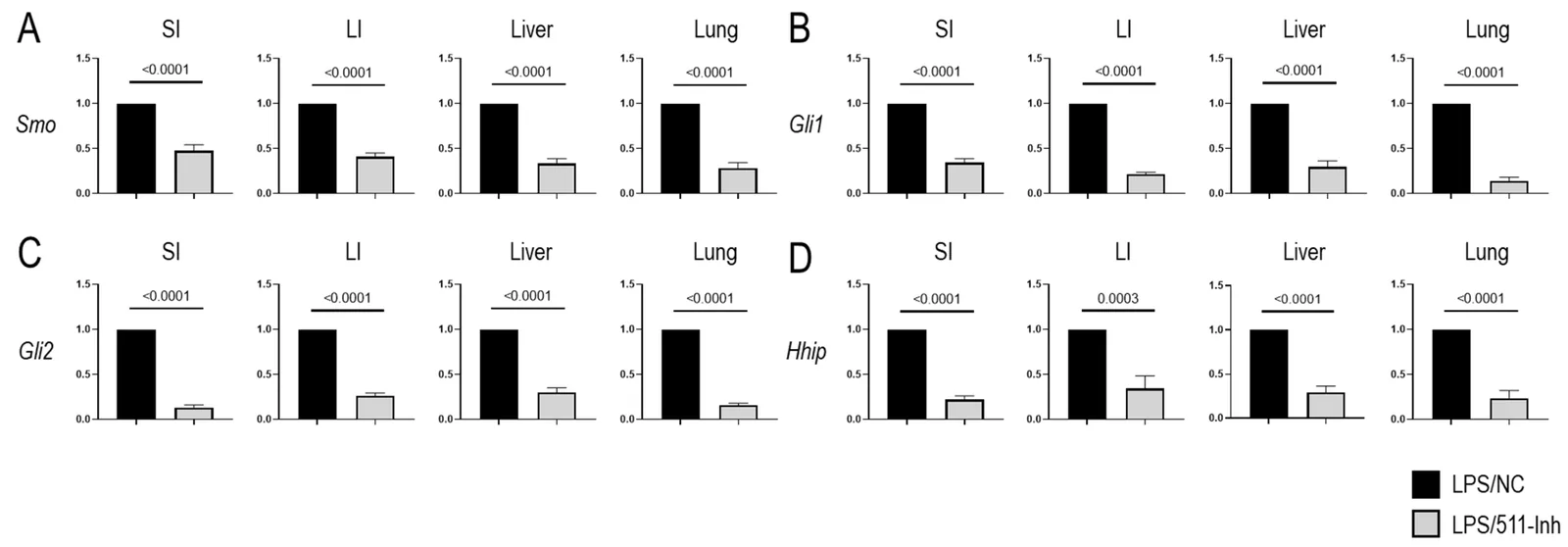
511-Inh treatment decreases expression of the hedgehog-related genes, including *Smo* (**A**), *Gli1* (**B**), *Gli2* (**C**), and *Hhip* (**D**) across multiple tissues. Relative gene expression levels in SI, LI, liver, and lung from LPS/NC or LPS/511-Inh mice. Levels of mRNA were quantified by RT-qPCR, normalized to *Actb*, and calculated using the 2^−ΔΔCT^ method relative to the control mean. Data represent mean ± SEM (*n* = 5–11 per group).

**Figure 9:**
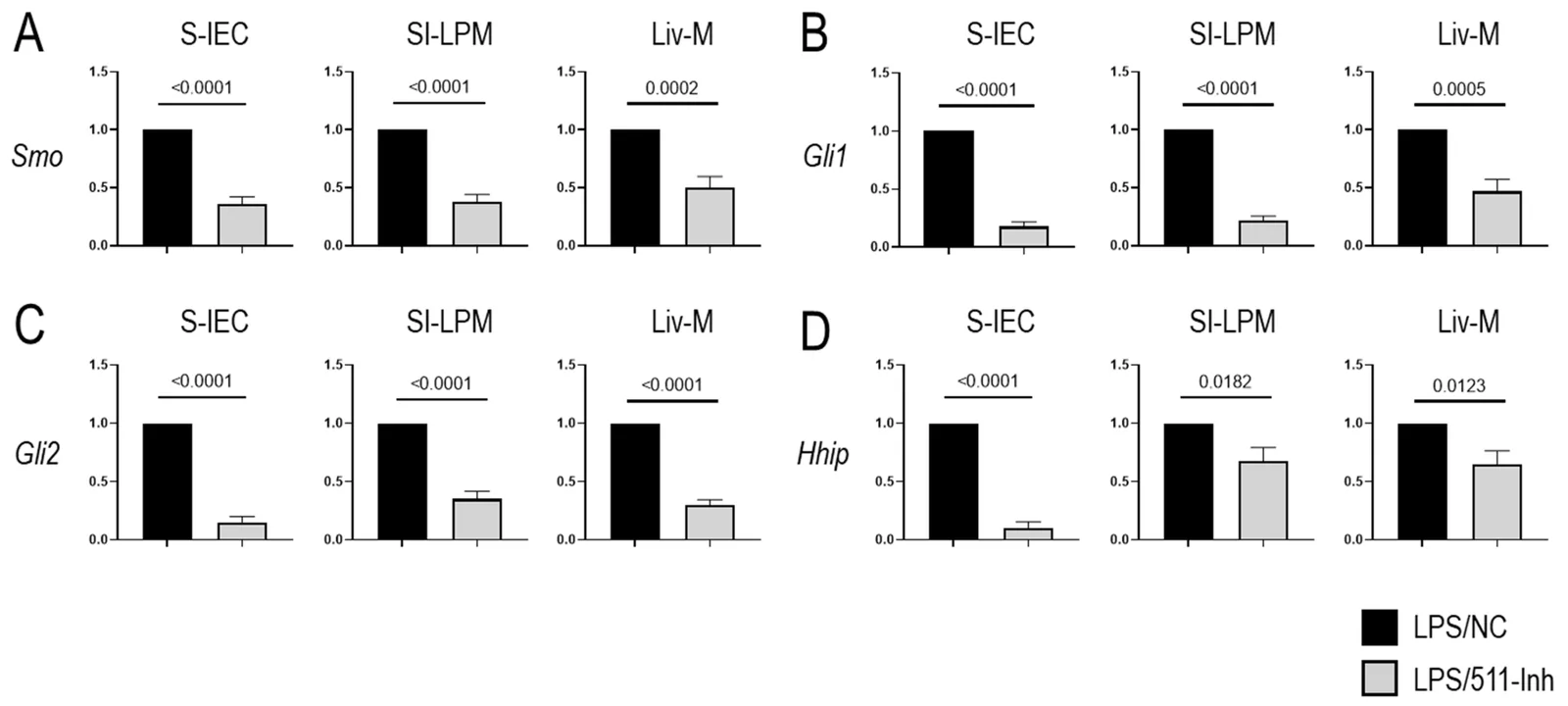
511-Inh treatment decreases expression of the hedgehog-related genes, including *Smo* (**A**), *Gli1* (**B**), *Gli2* (**C**), and *Hhip* (**D**) across multiple cell populations. Relative gene expression levels in S-IECs, SI-LPMs, and Liv-Ms from LPS/NC or LPS/511-Inh mice. Levels of mRNA were quantified by RT-qPCR, normalized to *Actb*, and calculated using the 2^−ΔΔCT^ method relative to the control mean. Data represent mean ± SEM (*n* = 5–11 per group).

We next investigated the expression of *Ptch1* altered by miR-511-3p knockdown, because it is a validated target of miR-511-3p and its gene protein serves as a negative regulator of the HH signaling pathway. miR-511-3p inhibition augmented *Ptch1* mRNA expression in multiple organs—including the SI, LI, liver, and lung (Figure 6A)—and the S-IECs, SI-LPMs and Liv-Ms of LPS-treated mice (Figure 6B). Compared to untreated controls, PTCH1 protein levels were considerably reduced in the SI, LI, liver, and lung tissues of NC-treated septic mice. These levels were effectively restored by miR-511-3p inhibition, with the most pronounced recovery observed in the SI and liver tissues (Figure 7 and Figure S8). To determine whether this PTCH1 upregulation suppresses HH signaling activity, we examined the expression of HH pathway-related genes. Consistent with these data, mRNA expression of *Smo*, *Gli1*, *Gli2*, and *Hhip* was downregulated across the examined tissues (Figure 8). Similar expression patterns were also observed in isolated S-IECs, SI-LPMs, and Liv-Ms (Figure 9). These results suggest that miR-511-3p inhibition reverses the sepsis-induced downregulation of PTCH1, thereby suppressing HH pathway activity in the indicated cell populations (S-IECs, SI-LPMs, Liv-Ms) and tissues (SI, LI, liver, lung).

To investigate the relationship between sepsis-induced miR-511-3p, its target—*PTCH1*—and proinflammatory cytokines in human intestinal epithelial cells, HCT116 cells were treated with LPS. LPS stimulation remarkably upregulated the expression of miR-511-3p, *IL6* and *TNF* while reducing the expression of *PTCH1* (Figure S5A). Transfection with a miR-511-3p antagomir markedly attenuated the LPS-induced upregulation of *IL6* and *TNF* and augmented *PTCH1* expression (Figure S5B). These results indicate that miR-511-3p inhibition mitigates inflammatory responses in human intestinal epithelial cells during sepsis, suggesting that this regulatory mechanism is conserved between murine and human epithelial cells.

Together, these findings demonstrate that miR-511-3p inhibition suppresses sepsis-induced systemic inflammation by restoring Ptch1 expression and attenuating Hedgehog (HH) signaling. This highlights the miR-511-3p/PTCH1/HH axis as a critical regulatory pathway in the pathogenesis of sepsis-related inflammation.

### 3.4. miR-511-3p Inhibition Ameliorates Sepsis-Induced Liver Injury

**Figure 10:**
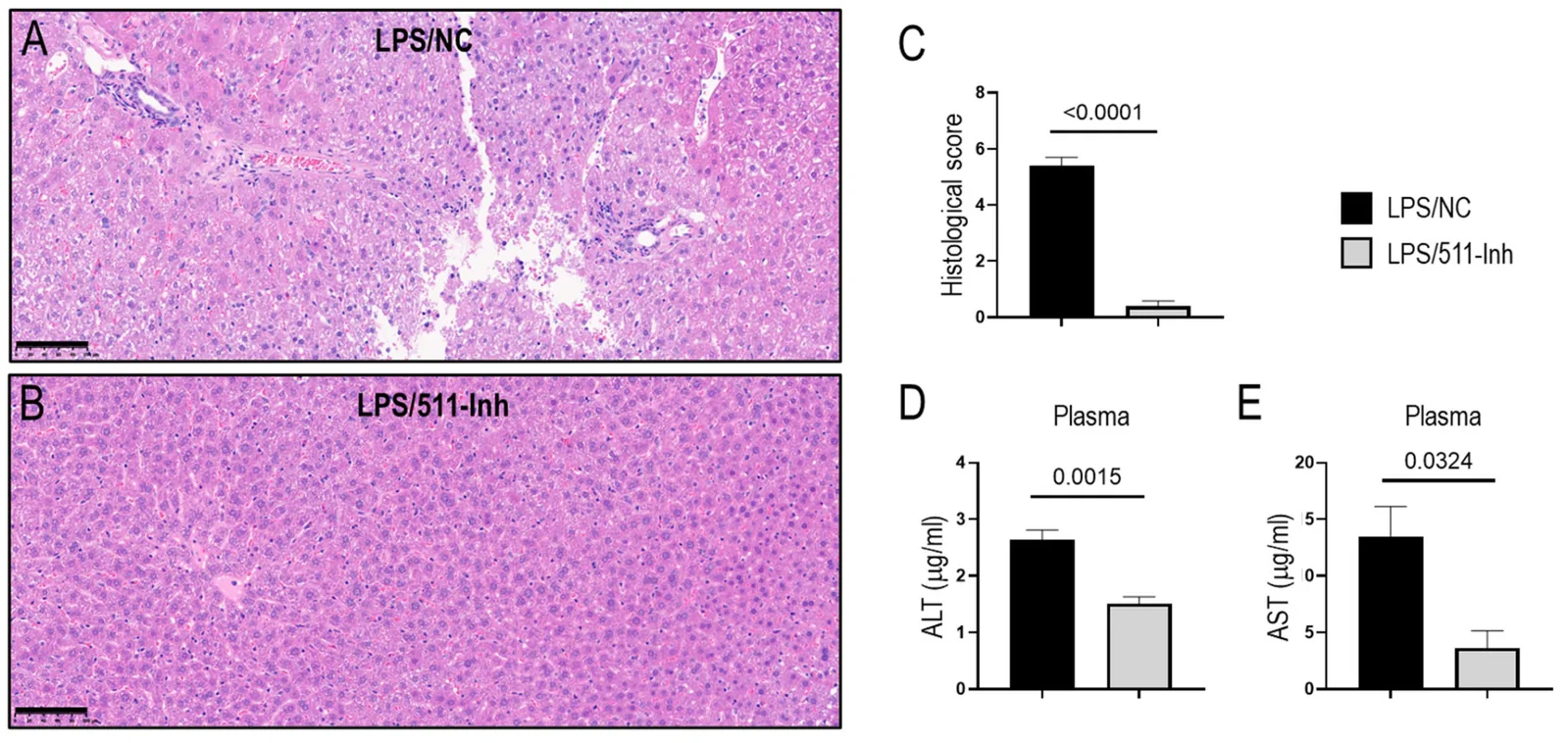
511-Inh treatment exhibits a protective effect on LPS-induced acute liver injury. (**A**,**B**) Representative hematoxylin and eosin (H and E)-stained liver sections from LPS/NC (**A**) or LPS/511-Inh mice (**B**). (**C**) Quantitative evaluation reveals a significant change in the histopathological score of liver damage in LPS/NC or LPS/511-Inh mice. (**D**,**E**) 511-Inh treatment lowers plasma concentrations of ALT (**D**) and AST (**E**) following LPS challenge. Data represent mean ± SEM (*n* = 3–5 per group). Scale bars, 100 μm.

Aberrant inflammation during sepsis frequently leads to multiple organ dysfunction syndrome. To evaluate the therapeutic potential of targeting miR-511-3p, we assessed the impact of its inhibition on organ injury in septic mice treated with either 511-Inh or NC. Histological examination revealed moderate liver tissue injury in the NC group, characterized by inflammatory cell infiltration, hepatocellular degeneration, and tissue disorganization (Figure 10A and Figure S9). In contrast, the inhibition of miR-511-3p remarkably ameliorated these pathological alterations, reducing inflammatory infiltrates and preserving normal hepatic architecture (Figure 10B and Figure S9). Consistent with these observations, histopathological scoring revealed a significant reduction in liver injury, decreasing from mild-to-moderate damage (5.4/16 points) to minimal damage (0.4/16 points) (Figure 10C and Table S3). Because plasma alanine aminotransferase (ALT) and aspartate aminotransferase (AST) levels are established indicators of liver injury [37], we assessed these parameters. Both ALT and AST levels were significantly lower in the septic mice treated with the 511-Inh than in those receiving NC (Figure 10D,E), consistent with reduced liver injury. No pathological differences were observed between the two cohorts in other tissues, including the SI (Figure S6A,B), LI (Figure S6C,D), lung (Figure S6E,F), and kidney (Figure S6G,H). This lack of variation likely stems from milder sepsis-induced damage in these organs compared to the liver under the current experimental conditions. Furthermore, while LPS-induced septic mice treated with NC exhibited lethargy, tactile unresponsiveness, and reduced mobility, 511-Inh treatment ameliorated these behavioral deficits. This behavioral improvement strongly correlates with the molecular and histological findings, particularly those observed in the liver.

### 3.5. Pharmacological Inhibition of Hedgehog Signaling Suppresses Inflammatory Responses in Septic Mice

Based on our current findings that sepsis-induced upregulation of miR-511-3p suppresses *Ptch1* expression and promotes HH signaling, we next investigated whether the pharmacological inhibition of HH signaling with vismodegib could attenuate septic inflammation. Vismodegib, an FDA-approved SMO inhibitor for cancer treatment [38], was administered (Figure S7A) to determine whether direct inhibition of HH signaling could reproduce the anti-inflammatory effects observed following miR-511-3p inhibition. Vismodegib treatment significantly reduced the expression of *Smo*, *Gli2* and *Hhip* in SI, LI, liver and lung tissues (Figure S7B–D). Moreover, this vismodegib’s effect was concurrent with a reduction in *Il6* and *Tnf* expression (Figure S7E,F). These effects were aligned with those of miR-511-3p inhibition, demonstrating that the pharmacological blockade of SMO recapitulated the simultaneous suppression of HH signaling and inflammation.

Taken together, our study supports a mechanistic model in which the sepsis-induced upregulation of miR-511-3p suppresses PTCH1 expression, thereby promoting SMO/GLI-mediated HH signaling and the expression of proinflammatory genes. Conversely, inhibition of miR-511-3p restores PTCH1 expression and attenuates HH signaling, resulting in reduced inflammatory responses and protection against sepsis-associated organ injury. The comparable effects of miR-511-3p inhibition and pharmacological SMO blockade further support the involvement of the PTCH1/SMO/GLI axis in sepsis-induced inflammation.

## 4. Discussion

Sepsis is characterized by dysregulated host inflammatory responses leading to tissue injury, organ dysfunction, and high mortality [39]. A number of cytokines, signaling pathways, and immune cell populations have been implicated in the pathogenesis of sepsis. However, the molecular mechanisms by which miRNAs regulate inflammatory signaling during sepsis remains incompletely understood. In the present study, we identified the miR-511-3p/PTCH1/HH signaling axis as an important regulator of systemic inflammation and multiple organ injury during sepsis. Acute sepsis upregulates miR-511-3p in target organs and immune cells, post-transcriptionally suppressing its direct target, *Ptch1*. Because PTCH1 negatively regulates Hedgehog (HH) signaling, its downregulation activates SMO and downstream GLI transcription factors, inducing pro-inflammatory mediators like IL-6 and TNF. Systemic miR-511-3p inhibition restored PTCH1 expression, reduced HH pathway activity, and attenuated systemic inflammation and liver injury. Pharmacological SMO blockade with vismodegib recapitulated these anti-inflammatory effects, confirming that aberrant HH signaling drives the inflammatory phenotype downstream of miR-511-3p. Thus, the miR-511-3p/PTCH1/HH axis is a crucial regulator of sepsis pathogenesis and a promising therapeutic target.

Our present data are consistent with previous studies demonstrating a proinflammatory role for HH signaling in multiple disease contexts. Activation of HH signaling has been reported to promote inflammatory cytokine production in epithelial cells, including A549 lung epithelial cells [25], while HH pathway activation in macrophages contributes to the progression of inflammatory liver disease in models of nonalcoholic fatty liver disease [40]. Together with these observations, our results support the concept that aberrant HH signaling functions as an important driver of inflammatory responses across diverse cell types and tissues in the context of an acute endotoxemia-induced sepsis model.

Our current findings extend our previous observations from a chronic sepsis model, in which miR-511-3p was identified as the only miRNA upregulated in both intestinal epithelial cells (IECs) and the circulation [33]. We demonstrate that miR-511-3p is also induced during acute endotoxemia-induced sepsis. This suggests that its dysregulation is a shared response to septic insults, rather than a phenomenon restricted to chronic sepsis. The elevated serum levels of miR-511-3p levels observed in patients with sepsis (Figure S1) further support its clinical relevance. The LPS-induced sepsis model identified miR-511-3p as an early responder. However, validation in another acute model is necessary. Specifically, a cecal ligation and puncture (CLP) will help evaluate its kinetics under living polymicrobial sepsis and therapeutic interventions, ultimately strengthening these findings. These results indicate that miR-511-3p is consistently associated with systemic inflammation in various sepsis models and clinical settings. This highlights its potential as a biomarker and mediator of sepsis pathophysiology.

The gastrointestinal tract is increasingly recognized as a critical driver, or “motor,” of systemic inflammation during sepsis [6]. Under physiological conditions, the intestinal epithelium maintains barrier integrity and immune homeostasis, thereby limiting the translocation of microorganisms and microbial products into the circulation [7]. During sepsis, the epithelial barrier is disturbed. This allows microbial products like LPS to spread throughout the body. These products can then trigger inflammation in distant organs such as the liver and lung [7]. Our results reveal a coordinated miR-511-3p response within the intestinal compartment during septic challenge. miR-511-3p expression was significantly increased in IECs and was accompanied by increased *Il6* and *Tnf* expression, suggesting that IECs participate in the local inflammatory response. Strikingly, CD11b^+^ small intestinal lamina propria macrophages (SI-LPMs) exhibited approximately 100-fold higher basal miR-511-3p expression than IECs (Figure S3), which was further increased during sepsis together with marked induction of *Il6* and *Tnf*. These findings highlight SI-LPMs as a particularly prominent cellular compartment for miR-511-3p expression during sepsis. miR-511-3p is encoded within intron 5 of the mannose receptor C-type 1 (Mrc1/CD206) gene and is co-expressed with CD206 in macrophages [34]. Because of this link, its high expression in SI-LPMs may reflect a specific immune response. Specifically, it likely indicates the activation or expansion of a macrophage-associated regulatory program during septic inflammation. The upregulation of miR-511-3p in SI-LPMs coincides with their pronounced inflammatory phenotype, suggesting that these cells may amplify intestinal inflammation and the gut–organ inflammatory axis during sepsis. Notably, liver macrophages (Liv-Ms) exhibited a similar pattern, characterized by increased miR-511-3p expression and activation of HH signaling during sepsis. These findings raise the possibility that coordinated macrophage responses in the intestine and liver participate in interorgan communication during septic inflammation. Therefore, further studies are warranted to elucidate the molecular mechanisms underlying macrophage-mediated gut–liver crosstalk in sepsis.

PTCH1 is a validated target of miR-511-3p and acts as the principal inhibitory receptor of the HH signaling pathway [22]. In the absence of hedgehog ligands, PTCH1 inhibits SMO activity, thereby preventing the nuclear translocation of GLI transcription factors and subsequent activation of HH target genes. Our findings suggest that the sepsis-associated upregulation of miR-511-3p disrupts this regulatory checkpoint, leading to the aberrant activation of HH signaling. We observed an inverse relationship between miR-511-3p and PTCH1 expression across multiple tissues. Reduced PTCH1 levels in the SI, LI, liver, and lung correlated with increased expression of HH pathway-related genes. Notably, dysregulation of these genes was also evident in purified IECs and CD11b^+^ macrophages, suggesting that the miR-511-3p-mediated disruption of PTCH1 signaling occurs across distinct cellular compartments during sepsis. Although the HH pathway has traditionally been recognized for its essential roles in embryonic development and tissue regeneration [23], accumulating evidence indicates that aberrant HH signaling can also modulate immune and inflammatory responses [41]. Our findings build on these observations. Excessive HH pathway activation may increase inflammation during acute sepsis. This process likely involves both canonical HH signaling and HH-independent mechanisms.

To establish the causal role of the miR-511-3p/PTCH1/HH signaling axis and evaluate its therapeutic potential, we employed two complementary approaches to target this axis. Systemic administration of the miR-511-3p-specific inhibitor effectively reduced the expression of the proinflammatory cytokines, IL-6 and TNF, and upregulated PTCH1 expression in the intestine and liver. These changes were accompanied by the suppression of key HH pathway components. This suggests that the inhibition of miR-511-3p alleviates sepsis-associated inflammation, partly by restoring the PTCH1-mediated repression of HH signaling. To further determine whether the anti-inflammatory effects of miR-511-3p inhibition were mediated through HH pathway suppression, we treated septic mice with the selective SMO inhibitor vismodegib. Pharmacological inhibition of SMO mirrored the effects of miR-511-3p inhibition. It reduced the expression of *Smo*, *Gli*, *Hhip*, *Il6*, and *Tnf* in IECs, macrophages, and sepsis-affected tissues. These matching results prove that the aberrant activation of the PTCH1/SMO/GLI axis drives sepsis-associated hyperinflammation. Consequently, this pathway represents a promising therapeutic target.

At the organ level, the effects of miR-511-3p inhibition were particularly pronounced in the liver. Treatment with 511-Inh significantly attenuated sepsis-associated liver injury, accompanied by reduced inflammatory cell infiltration, diminished hepatocellular degeneration, and improved preservation of hepatic architecture. These histopathological findings were consistent with the observed molecular changes, further supporting a pathogenic role for miR-511-3p in hepatic inflammation during sepsis. In contrast, no significant histopathological differences were detected in the lungs or kidneys between control- and 511-Inh-treated septic mice at 24 h after sepsis induction. These findings suggest that the liver may be particularly susceptible to miR-511-3p/PTCH1-axis-mediated inflammatory injury during the early phase of sepsis. Alternatively, the lack of detectable changes in the lung and kidney may reflect differences in the kinetics or severity of tissue injury across organs. Collectively, these results highlight the temporal and organ-specific regulation of the miR-511-3p/PTCH1 axis during sepsis progression and identify the liver as a potential primary target of miR-511-3p-mediated inflammatory signaling. Furthermore, it would be valuable to compare our findings with other HH signaling-related miRNAs, such as miR-326, miR-9, and miR-130a-5p [42,43,44], under septic conditions in future studies.

The present study has several limitations. First, although our findings strongly support PTCH1 as a functional target of miR-511-3p, further investigation—such as PTCH1 rescue experiments or conditional genetic manipulation of HH pathway components—is warranted to definitively establish causality. In particular, future *Ptch1* loss-of-function studies will be essential to establish its causal and therapeutic roles in sepsis. Second, this study relied primarily on an LPS-induced sepsis model, which recapitulates key inflammatory features of sepsis but does not fully reflect the complexity of human disease. The short 24 h observation period further limits the generalizability of the findings. Validation in clinically relevant polymicrobial sepsis models will be necessary to establish the translational significance of these findings. Third, due to resource constraints and limitations of the experimental setup, several assessments, such as survival rate and functional barrier assays, could not be performed. These limitations should be addressed in future studies to provide a more comprehensive evaluation. Fourth, the relatively small sample size, combined with the absence of formal normality testing and an a priori power analysis, may limit the robustness and generalizability of our findings. Additionally, the zero variance in certain control groups complicated data distribution and the selection of appropriate statistical tests. Consequently, future validation in larger cohorts employing rigorous statistical methods and a formal power analysis is warranted. Fifth, because our findings regarding HH pathway-related molecules (SMO, GLI1, GLI2, and HHIP) rely primarily on mRNA-level data, further validation using optimized or alternative protein detection methods remains necessary. Sixth, the cellular sources and mechanisms responsible for the release of circulating miR-511-3p remain to be determined. Given the higher expression of miR-511-3p in macrophages, future investigations should assess whether the extracellular vesicle-mediated transfer of miR-511-3p contributes to systemic inflammation and intercellular communication during sepsis.

## 5. Conclusions

In this study, we have identified the miR-511-3p/PTCH1/HH signaling axis as a novel and critical regulator of systemic inflammatory responses during sepsis. Our results indicate that targeting this axis—either by inhibiting the upstream miRNA or by pharmacologically blocking the downstream SMO receptor—represents a potent therapeutic strategy to suppress dysregulated host inflammation and protect against multiple organ injury. Crucially, these findings bridge the gap between molecular discovery and clinical application, offering a promising framework for developing targeted, bench-to-bedside immunomodulatory therapies for critically ill patients. Future studies should investigate the long-term systemic effects of these interventions in chronic or polymicrobial sepsis models to further pave the way toward clinical development.

## Data Availability

The original contributions presented in this study are included in the article/Supplementary Material. Further inquiries can be directed to the corresponding author (E.J.P.).

## Supplementary Materials

The following supporting information can be downloaded at https://www.mdpi.com/article/10.3390/cells15212009/s1: Figure S1. Validation of hsa-miR-511-3p expression in clinical serum samples; Figure S2. LPS treatment increased expression of miR-511-3p (A), *Il6* (B) and *Tnf* (C) across multiple tissues. Figure S3. Validation of miR-511-3p expression in S-IECs and SI-LPMs; Figure S4. LPS treatment decreased *Ptch1* (A) and increased the Hedgehog-related genes, including *Smo* (B), *Gli1* (C), *Gli2* (D) and *Hhip* (E) across multiple tissues; Figure S5. LPS treatment increased miR-511-3p, *IL6*, and *TNF* and decreased *PTCH1* expression in HCT116 cells (A), and miR-511-3p antagomir (511-Ant) transfection reversed their expressions in LPS-treated cells (B); Figure S6. Representative hematoxylin and eosin (H and E) staining images of the SI (A,B), LI (C,D), lung (E,F) and kidney (G,H) from LPS-induced septic mice treated with either a NC RNA (A,C,E,G) or a 511-Inh RNA (B,D,F,H) are shown; Figure S7. Vismodegib treatment (as shown in A) decreased the Hedgehog-related genes, including *Smo* (B), *Gli2* (C), and *Hhip* (D) and proinflammatory cytokine genes, *Il6* (E) and *Tnf* (F), across multiple tissues of LPS-induced septic mice; Figure S8. Uncropped Western blot images; Figure S9. Liver tissue images used for histological scoring; Table S1. Hematological profiles of patients with sepsis; Table S2. Primer sequences used in RT-qPCR; Table S3. Histopathological scores of liver tissues.
