# Supplementary material for: Inhibiting the miR-511-3p/PTCH1/Hedgehog Axis Alleviates Sepsis-Induced Systemic Inflammation and Acute Liver Injury
Source: Cells. 2026 Nov 4;15(21):2009. doi: 10.3390/cells15212009 (PMC13761787; doi:10.3390/cells15212009)
Supplement: Supplementary file 1 [file cells-15-02009-s001.zip › cells-4605674-supplementary.pdf]

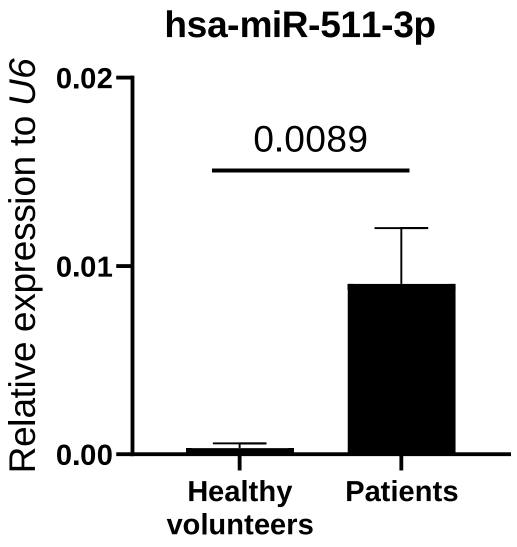

Figure S1. Validation of hsa-miR-511-3p expression in clinical serum samples. Relative expression of hsa-miR-511-3p in serum samples was analyzed by RT-qPCR and normalized to *U6* small nuclear RNA (*U6*) ($2^{-\Delta CT}$). Data are expressed as mean ± SEM (n = 10); *p*-value is displayed on graph, where $p < 0.05$ indicates a statistical significance.

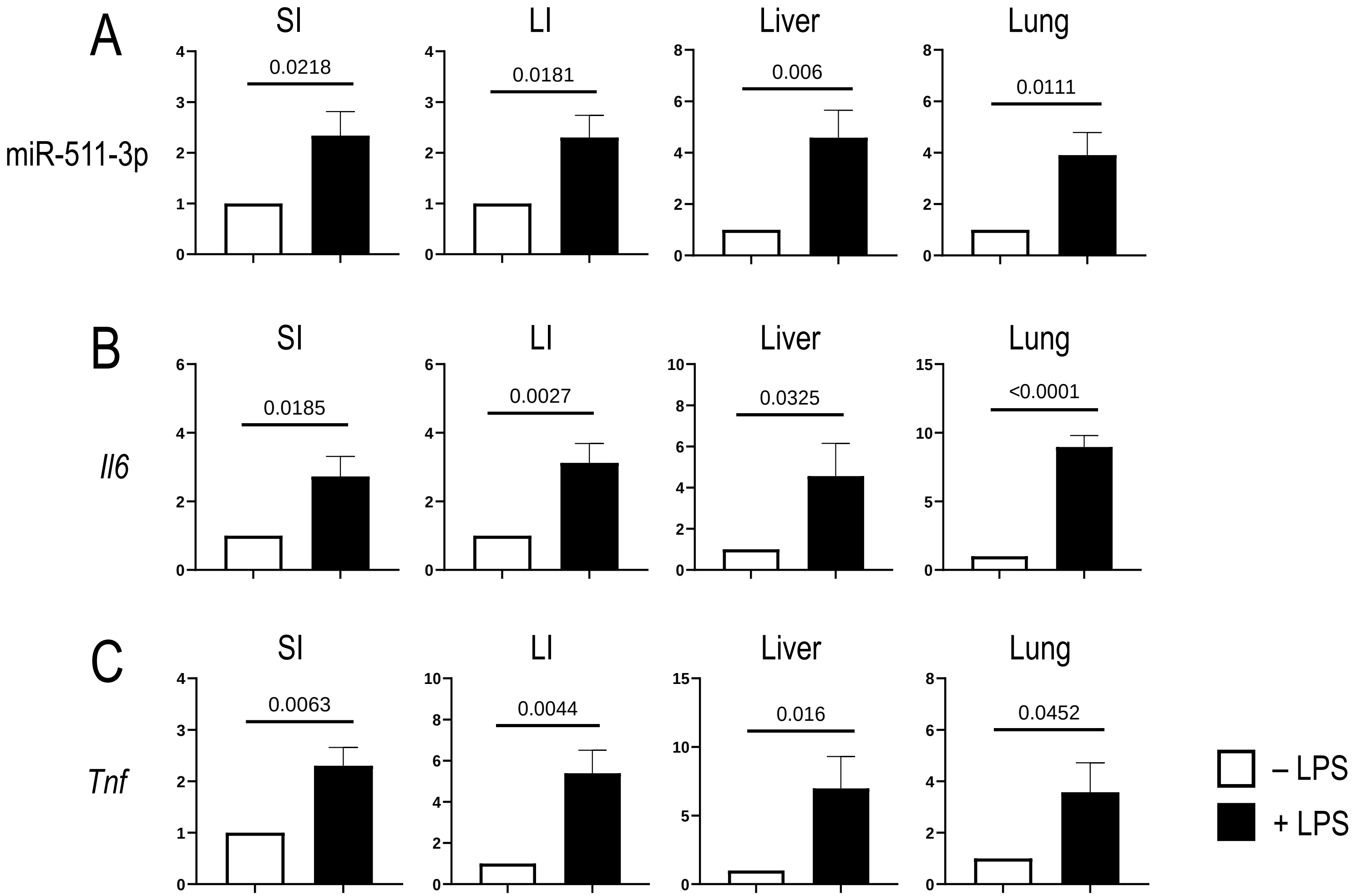

Figure S2. LPS treatment increases expressions of miR-511-3p (**A**), *Il6* (**B**) and *Tnf* (**C**) across multiple tissues. Relative gene expression levels in small intestine (SI), large intestine (LI), liver and lung from control (- LPS) and LPS-treated (+ LPS) mice. Levels of miRNA and mRNA were quantified by RT-qPCR, normalized to *U6* and *Actb* (b-actin), respectively, and calculated using the $2^{-\Delta\Delta CT}$ method relative to the control mean. Data represent mean ± SEM (n = 5-8 per group); p-values are displayed on all graphs, where $p < 0.05$ indicates a statistical significance. *Il6*, interleukin 6 gene; and *Tnf*, tumor necrosis factor gene.

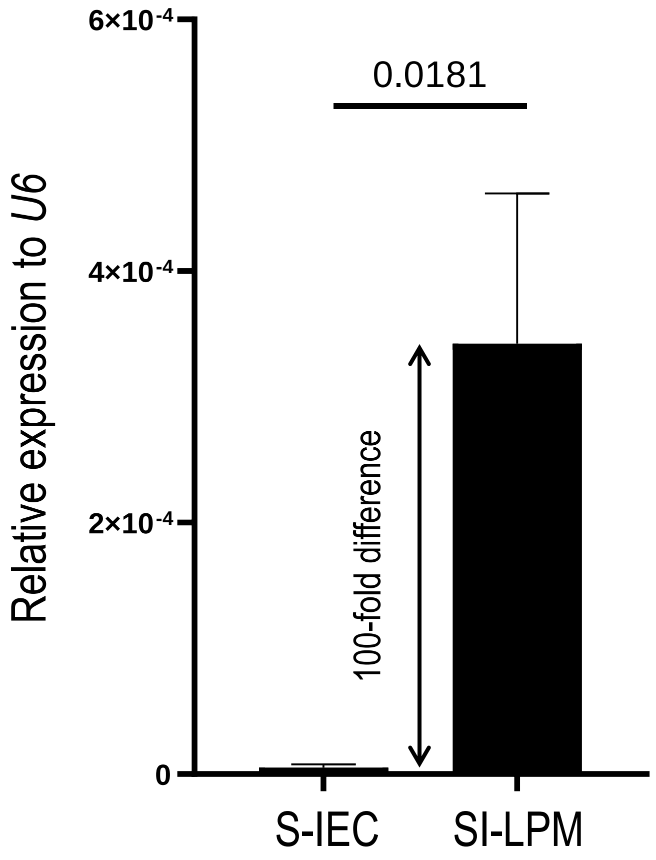

Figure S3. Validation of miR-511-3p expression in S-IECs and SI-LPMs. Relative expression of miR-511-3p was analyzed by RT-qPCR and normalized to *U6* small nuclear RNA (*U6*) ($2^{-\Delta CT}$). Data represent mean ± SEM (n = 6). S-IECs, small intestinal epithelial cells; and SI-LPMs, small intestinal lamina propria macrophages.

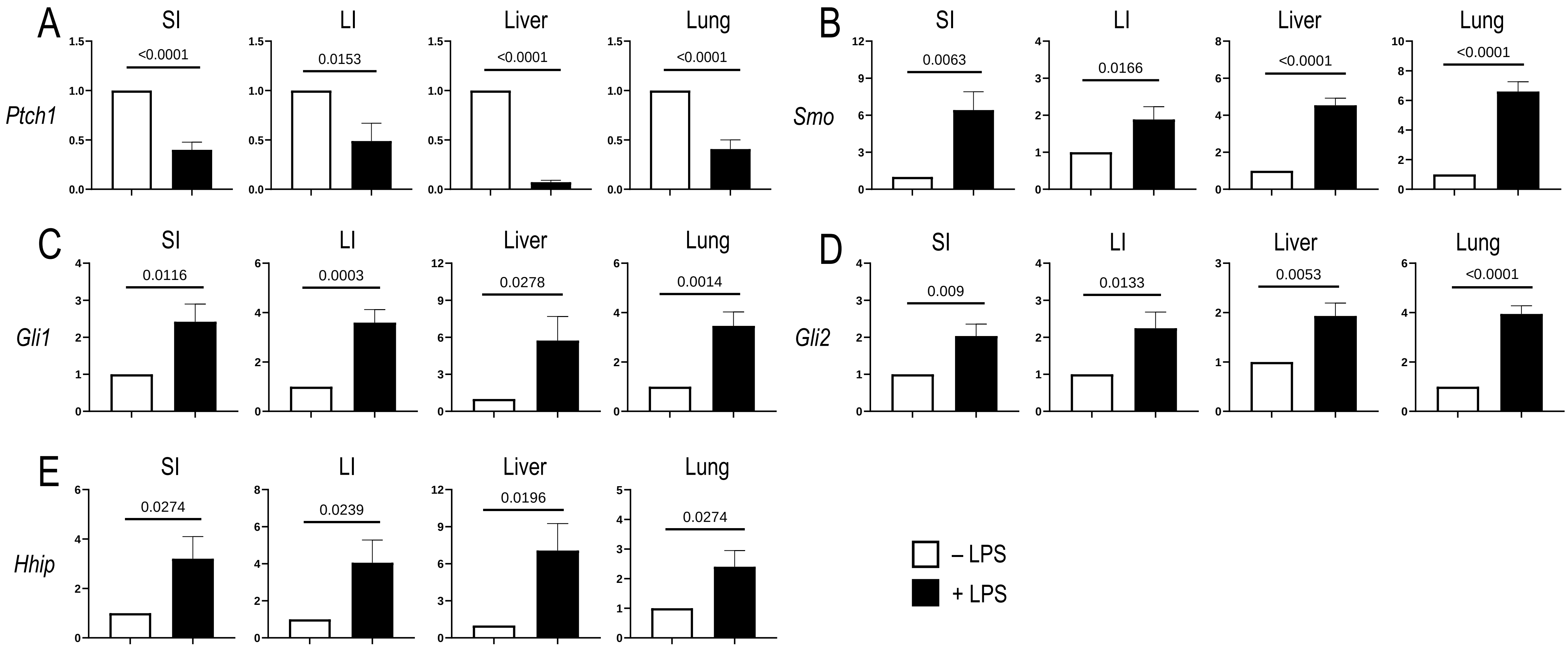

Figure S4. LPS treatment decreased expressions of *Ptch1* (**A**) and increased the Hedgehog-related genes, including *Smo* (**B**), *Gli1* (**C**), *Gli2* (**D**) and *Hhip* (**E**) across multiple tissues. Relative gene expression levels in S-IECs from control (- LPS) and + LPS mice. Levels of mRNA were quantified by RT-qPCR, normalized to *Actb*, and calculated using the $2^{-\Delta\Delta CT}$ method relative to the control mean. Data represent mean ± SEM (*n* = 5-11 per group).

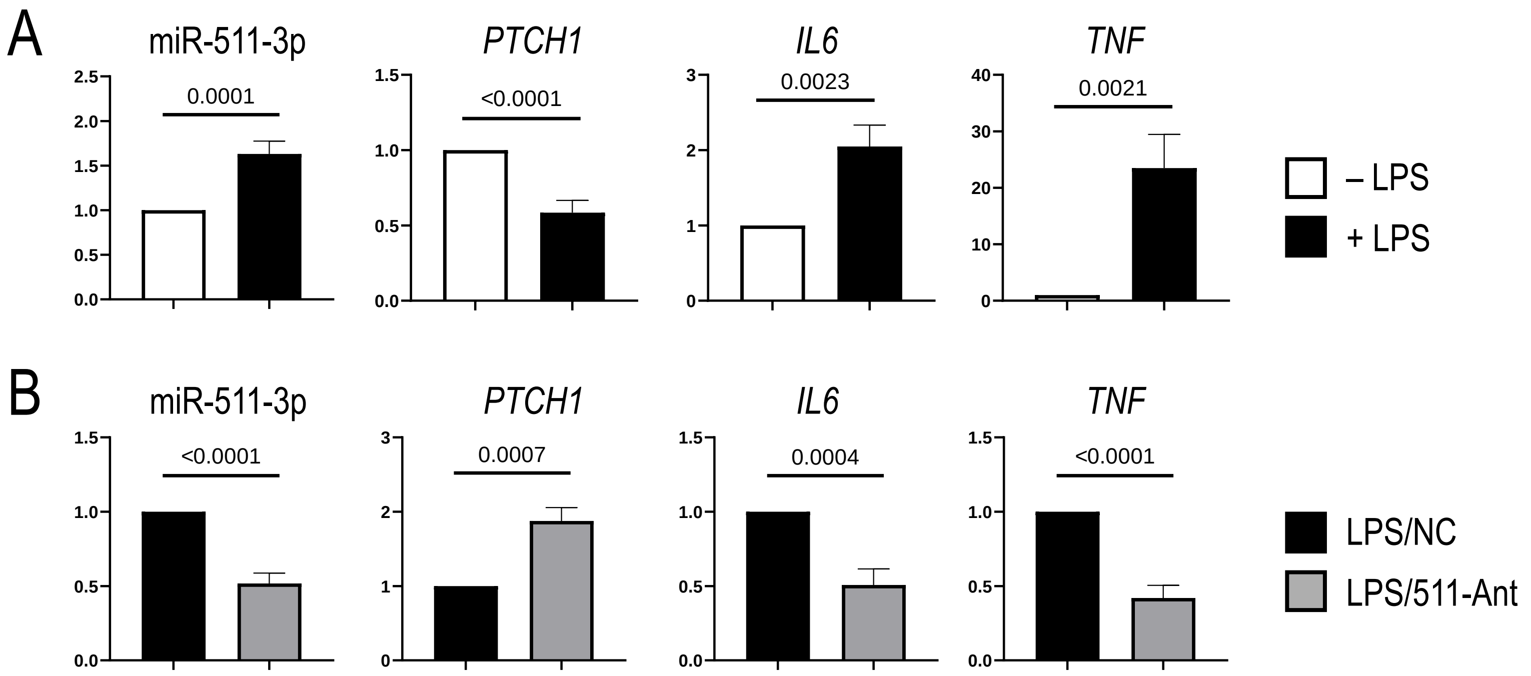

Figure S5. LPS treatment increased miR-511-3p, *IL6*, and *TNF* and decreased *PTCH1* expression in HCT116 cells (**A**), and miR-511-3p antagomir (511-Ant) transfection reversed their expressions in LPS-treated cells (**B**). Relative gene expression levels in control (- LPS) or LPS-treated (+ LPS) cells (**A**) and negative control (NC)-transfected (NC) or miR-511-3p antagomir (511-Ant) transfected LPS-treated cells (**B**). Levels of miRNA and mRNA were quantified by RT-qPCR, normalized to *U6* and *Actb*, respectively, and calculated using the $2^{-\Delta\Delta CT}$ method relative to the control mean. Data represent mean ± SEM (*n* = 6-10 per group). 511 Ant, miR-511-3p antagomir.

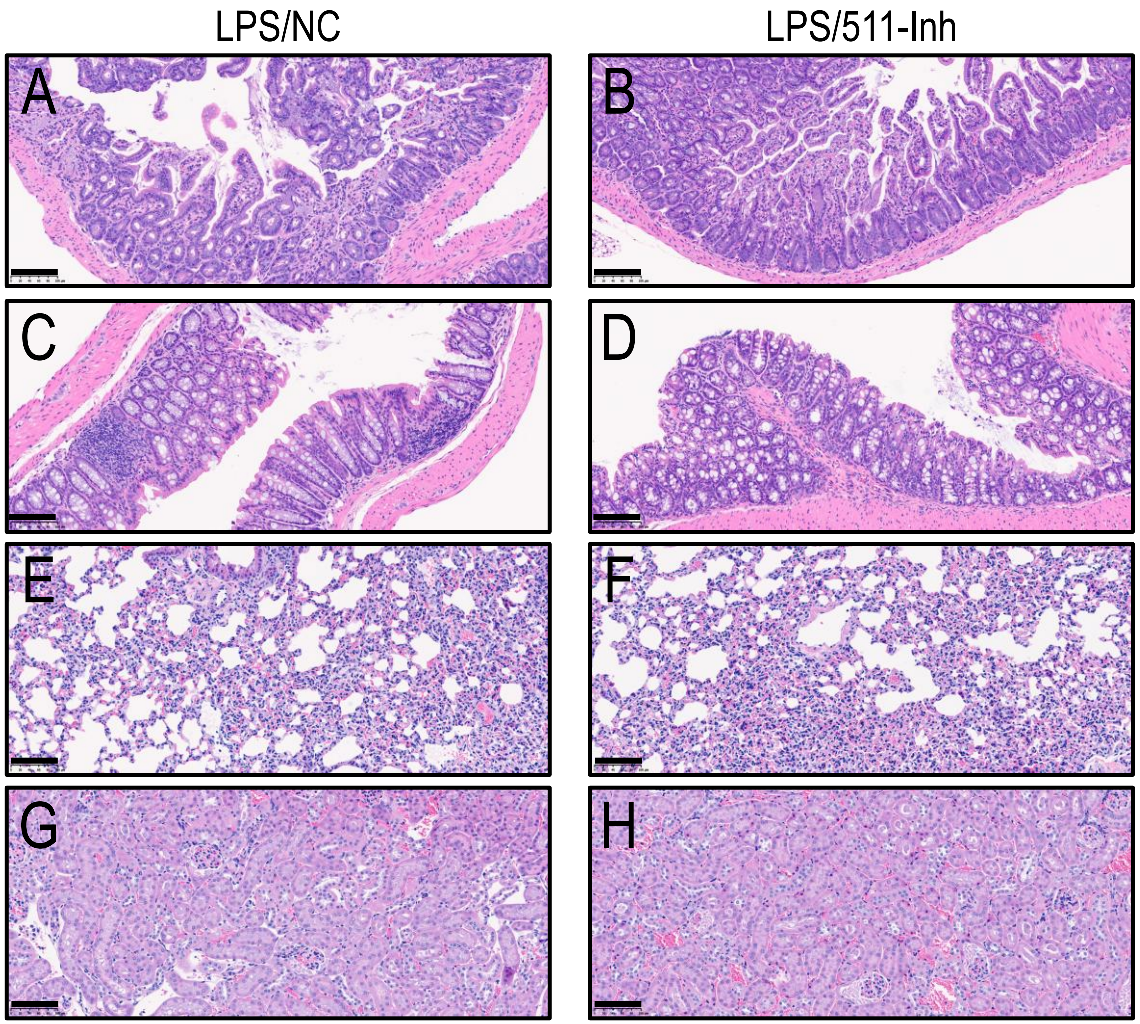

Figure S6. Representative hematoxylin and eosin (H and E) staining images of the SI (**A**,**B**), LI (**C**,**D**), lung (**E**,**F**), and kidney (**G**,**H**) from LPS-induced septic mice treated with either a NC RNA (**A**,**C**,**E**,**G**) or a 511-Inh RNA (**B**,**D**,**F**,**H**) are shown. Scale bars, 100 μm.

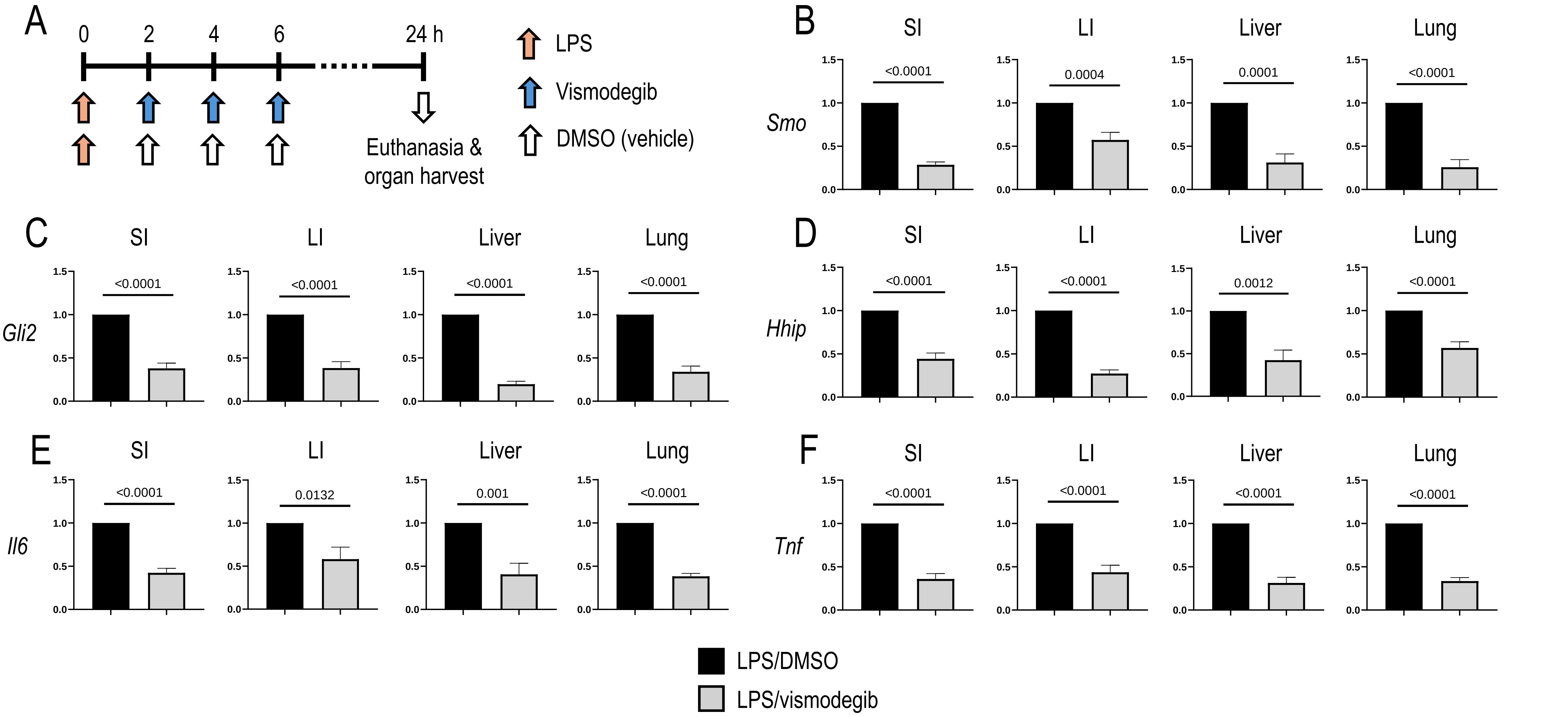

Figure S7. Vismodegib treatment (as shown in **A**) decreased the Hedgehog-related genes, including *Smo* (**B**), *Gli2* (**C**), and *Hhip* (**D**) and proinflammatory cytokine genes, *Il6* (**E**) and *Tnf* (**F**), across multiple tissues of LPS-induced septic mice. (A) Experimental design timeline. (B-F) Relative gene expression levels in SI, LI, liver, and lung from LPS-induced sepsis mice treated with vehicle (LPS/DMSO) or vismodegib (LPS/vismodegib). Levels of mRNA were quantified by RT-qPCR, normalized to *Actb*, and calculated using the $2^{-\Delta\Delta CT}$ method relative to the control mean. Data represent mean ± SEM (*n* = 5-7 per group).

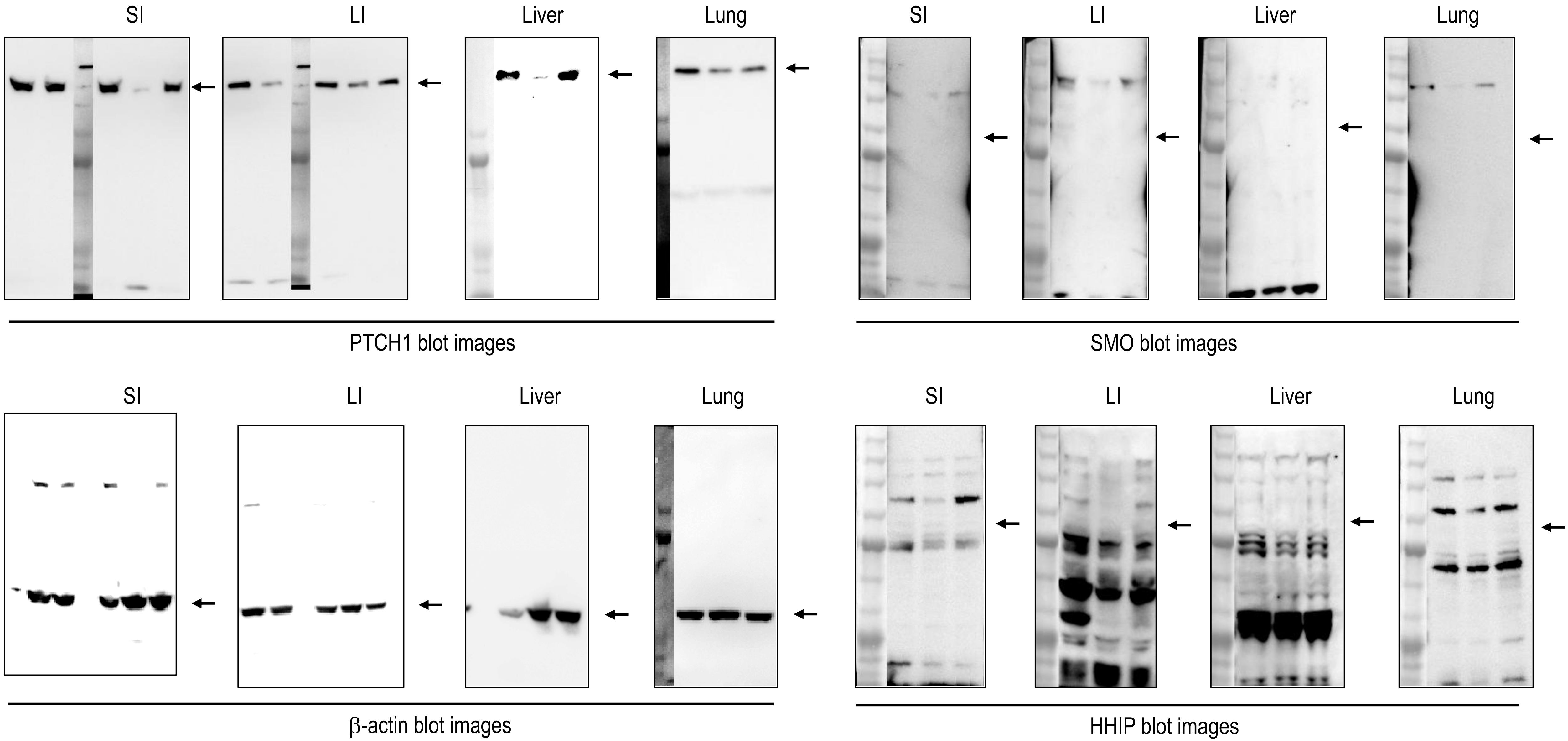

Figure S8. Uncropped Western blot images

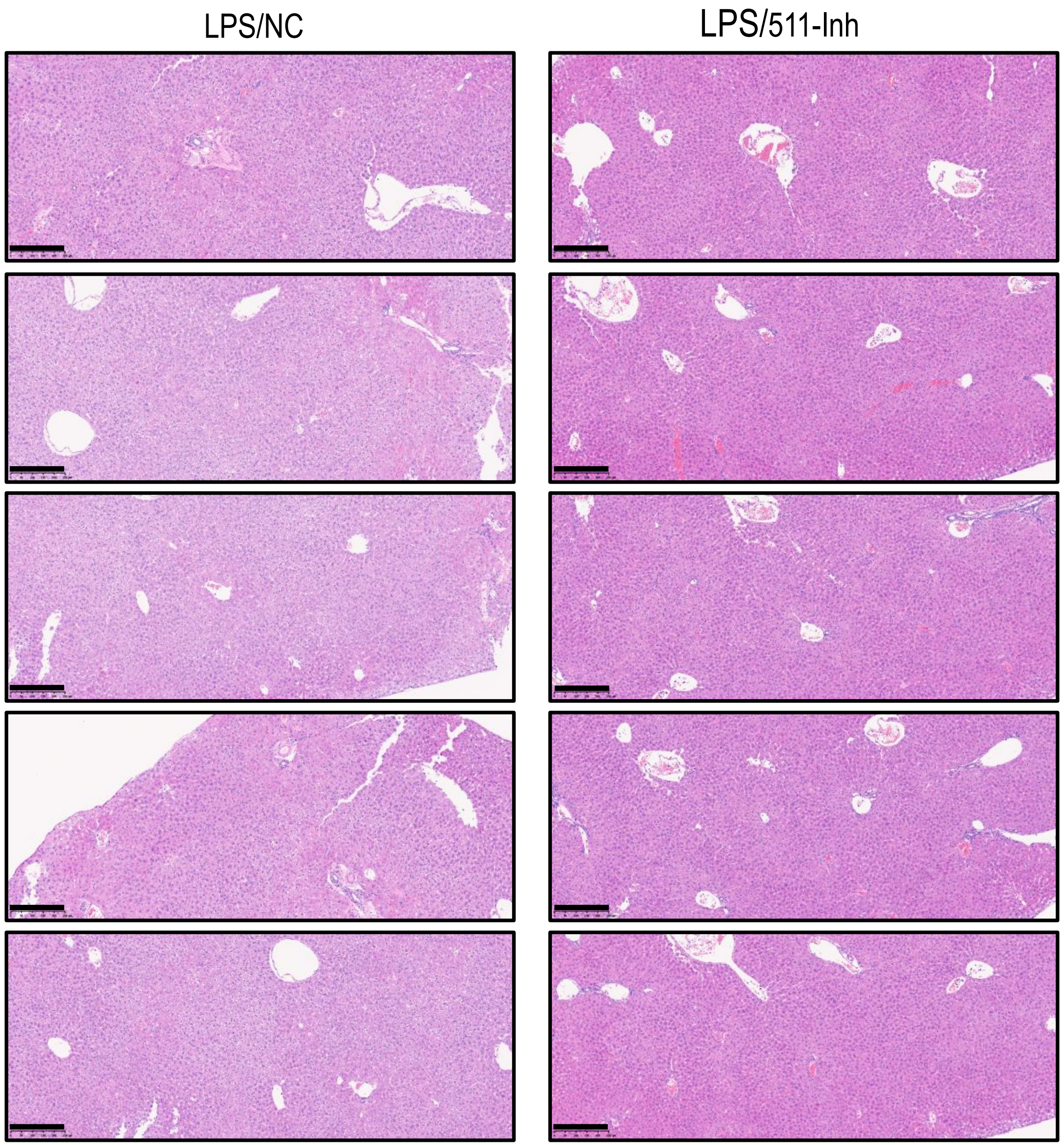

Figure S9. Liver tissue images used for histological scoring. Scale bars, 200 µm.

Table S1. Hematological profiles of patients with sepsis

ID	Disorder	Age	Sex	SOFA score	GCS	Death (1)	HR	sBP	dBP	FiO2
1	Urinary Tract Infection	70	M	3	11	1	109	140	57	0.25
2	Acute Cholangitis	70	F	6	10	0	85	175	69	0.35
3	Pneumonia、ARDS	87	M	4	14	0	86	122	47	0.55
4	Pneumonia	95	M	9	3	0	76	72	31	0.4
5	Pneumonia	59	F	8	3	0	111	99	55	0.4
6	Herpes simplex encephalitis	82	M	5	7	0	119	107	52	0.4
7	Pneumonia	70	M	13	14	1	121	89	37	0.8
8	Pneumonia	80	F	7	6	0	80	98	43	0.4
9	Emphysematous Cystitis	86	M	5	14	0	104	128	66	0.25
10	Pneumonia	92	M	4	14	1	124	99	42	0.5

	Average			6.4	9.6	0.3	101.5	112.9	49.9	0.43
	SD			2.99	4.55	0.48	18.17	29.42	12.23	0.16
	SEM			0.95	1.44	0.15	5.75	9.30	3.87	0.05

BT	RR		TP	alb	BUN	Cre	eGFR	Na	K	Cl	Ca	AST
37.6	17		6.7	2.8	49.3	1.27	44.3	152	4	120	9.1	28
37.1	25		5	2.2	37.5	0.92	46.6	142	3.2	113	7.3	44
38	25		4.9	1.7	46.3	1.04	51.8	147	3.2	117	8.6	110
37.1	18		6.9	2.1	42.5	1.76	28.4	134	4.8	99	8.7	22
37.2	26		5.6	2.7	17.9	1.61	26.6	136	3.7	106	7.9	40
39	25		6.1	2.3	25.1	0.64	89.6	129	3.6	96	7.5	208
35	24		5.1	1.4	82.4	3.14	16.4	136	3.6	104	7.9	97
38.1	20		5.2	1.6	28.4	0.29	159.1	140	4	105	8.1	24
37.1	16		7.1	3.6	38.7	4.3	11	138	4.2	105	8.7	17
37.8	30		5.1	1.8	16.7	0.66	84	132	3.5	100	7.3	29

37.4	22.6		5.77	2.22	38.48	1.563	55.78	138.6	3.78	106.5	8.11	61.9
1.04	4.58		0.86	0.67	19.16	1.25	44.73	6.95	0.49	7.85	0.64	60.52
0.33	1.45		0.27	0.21	6.06	0.40	14.15	2.20	0.15	2.48	0.20	19.14

ALT	LDH	T-bil	Glu	CPK	CRP	WBC	RBC	Hb	Hct	Plt	APTT	PT
20	260	0.6	122	422	5.34	13370	339	9.8	30.7	303	24.7	12.3
29	346	1	189	776	32.5	16240	381	10	32.3	86	44.7	19.5
73	234	1.3	119	6	9.14	3940	311	9.8	29.7	184	32.8	15.9
8	142	0.4	131	25	20.08	11330	275	7.9	24.4	185	26.1	12.7
16	209	0.5	219	1251	6.61	1860	368	11.3	34.2	163	34.2	12.5
234	426	1	220	450	19.03	8520	324	11	31.6	76	70	17.8
66	195	0.7	95	80	24.84	3580	293	10.8	29.9	25	93.6	18.2
13	166	0.3	319	27	9.27	9400	261	7.5	24.2	115	46.8	12
4	266	0.7	133	264	4.01	21780	370	10.7	32.6	201	32	12.3
17	291	0.7	102	53	14.75	7740	306	9.1	27.7	225	32.7	13.2

48	253.5	0.72	164.9	335.4	14.557	9776	322.8	9.79	29.73	156.3	43.76	14.64
69.47	85.50	0.30	70.90	408.67	9.40	6166.10	41.28	1.29	3.37	81.79	21.93	2.91
21.97	27.04	0.10	22.42	129.25	2.97	1950.06	13.05	0.41	1.07	25.87	6.94	0.92

PT-INR	Fibrinogen	d-dimer	pH	CO2	O2	HCO3-	Lac
1.08	507	3.7	7.44	30.2	148.2	20.1	1.1
1.8	649	1.74	7.488	29.3	81.8	21.7	2.1
1.4	598	8.86	7.516	32.7	65.6	25.9	1.5
1.12	412	9.13	7.394	38.6	134.5	23.1	1.3
1.1	314	2.27	7.268	51.5	59.2	23	2.2
1.57	470	4.58	7.484	36.2	165.1	26.6	1.7
1.6	512	12.54	7.374	34.3	51.7	19.6	1.8
1.03	614	1.96	7.463	41.2	89.7	28.8	1
1.04	340	3.02	7.421	32	85.9	20.3	1.3
1.12	523	16.72	7.505	33.6	80.6	25.9	1.1

1.286	493.9	6.452	7.4353	35.96	96.23	23.5	1.51
0.28	112.54	5.14	0.08	6.57	39.16	3.16	0.43
0.09	35.59	1.63	0.02	2.08	12.39	1.00	0.13

Table S2. Primer sequences used in RT-qPCR

	Forward (5' → 3')	Reverse (5' → 3')
mmu-miR-511-3p	AATGTGTAGCAAAAGACAGGAT	Universal primer
mouse Ptch1	GTTGCAGCGTTAAAGGAA	CCTTCCCCTTGGAAATCTGCT
mouse Smo	GAGGCTACTTCCTCATCAGAGG	GCTGAAGGTGATGAGCACAAAGC
mouse Gli1	CTCAAACTGCCCAGCTTAACCC	TGCGGCTGACTGTGTAAGCAGA
mouse Gli2	ACACTGTGGAGGACTGCCTACA	GGCATCTCCATGCCACTGTCAT
mouse Hhip	CAACCAGGAACGGTGGGCTATT	TCTGCGACTTCCAGAAACACCC
mouse Il6	TGGAGTCACAGAAGGAGTGGCTAA[illegible]	TCTGACCACAGTGAGGAATGTCCAC
mouse Tnf	ATAAGTGTGAGCATGTATATGT	ATAGCTCCCAGAAAAGCAAGC
mouse Actb	GAAGTTGCCCATGTTATTTTTCG	CACCCCGAAGTTCAGTAGACA
hsa-miR-511-3p	AATGTGTAGCAAAAGACAGA	Universal primer
human PTCH1	GTTGCAGCGTTAAAGGAA	CCAGCGGCTACTTACTCA
human IL6	AATTCGGTACATCCTCGACGG	GGTTGTTTTCTGCCAGTGCC
human TNF	GACAAGCCTGTAGCCCATGTTGTA	TCTCAGCTCCACGCCATTG
human ACTB	CGATCCACACGGAGTACTTG	GATGCAGAAAGGAGATCACTG
U6	GCGCGTCGTGAAGCGTTC	GTGCAGGGTCCGAGGT

Table S3. Histopathological scores of liver tissues

	Congestion	Vacuolization	Necrosis	Inflammation	Sum
LPS/ NC (#1)	1	1	1.5	2	5.5
LPS/ NC (#2)	1.5	1	1	2.5	6
LPS/ NC (#3)	0.5	1	1.5	2	5
LPS/ NC (#4)	1	1	1.5	2.5	6
LPS/ NC (#5)	0.5	1	1	2	4.5
LPS/511-Inh (#1)	0	0	0	0	0
LPS/511-Inh (#2)	0	0.5	0	0.5	1
LPS/511-Inh (#3)	0	0	0.5	0	0.5
LPS/511-Inh (#4)	0	0	0	0.5	0.5
LPS/511-Inh (#5)	0	0	0	0	0
